# Localizing Tortoise Nests by Neural Networks

**DOI:** 10.1371/journal.pone.0151168

**Published:** 2016-03-17

**Authors:** Roberto Barbuti, Stefano Chessa, Alessio Micheli, Rita Pucci

**Affiliations:** Department of Computer Science, University of Pisa, Pisa, Italy; Georgia State University, UNITED STATES

## Abstract

The goal of this research is to recognize the nest digging activity of tortoises using a device mounted atop the tortoise carapace. The device classifies tortoise movements in order to discriminate between nest digging, and non-digging activity (specifically walking and eating). Accelerometer data was collected from devices attached to the carapace of a number of tortoises during their two-month nesting period. Our system uses an accelerometer and an activity recognition system (ARS) which is modularly structured using an artificial neural network and an output filter. For the purpose of experiment and comparison, and with the aim of minimizing the computational cost, the artificial neural network has been modelled according to three different architectures based on the input delay neural network (IDNN). We show that the ARS can achieve very high accuracy on segments of data sequences, with an extremely small neural network that can be embedded in programmable low power devices. Given that digging is typically a long activity (up to two hours), the application of ARS on data segments can be repeated over time to set up a reliable and efficient system, called Tortoise@, for digging activity recognition.

## 1 Introduction

The impact of the species extinction is of greater devastation than was ever expected [1]. Reptiles and amphibians seem to have been disproportionately affected, showing average population decreases of 34% and 97% respectively. The biggest threat to biodiversity historically comes from a combined impact of habitat loss, degradation, new predators, food, and the mistreatment of animals for material assets. This level and type of endangerment was reported in [2] and [3]. Given this history, the new main factor for population decline is the stark lack of diversity itself. The decrease in tortoise population is attributable to a combination of infertility and predation of eggs (at incubation, as well as young tortoises in their first years of life). The eggs are affected by environmental pollution, which inhibits hatching. The hatchlings are also easy prey for predators.

In an attempt to limit the tortoises mortality, herpetologists from many organizations have taken part in protection programs in order to monitor and support female tortoises and their hatchlings. In the Galapagos Islands, the Darwin Scientific Foundation and the Charles Darwin Foundation have been committed to protect giant tortoises (*Chelonoidis nigra*) [4]. In Jumby Bay, the population of hawksbill tortoises (Eretmochelis imbricata) is monitored by the Jumby Bay Hawksbill Project run by the Jumby Bay Company (*Eretmochelis imbricata*) is monitored by the Jumby Bay Hawksbill Project run by the Jumby Bay Company [5]. The Turtle Conservancy works to protect the population of gopher tortoises (*Gopherus polyphemus*) in southern California, and various species of turtles and tortoises in Madagascar and Mexico [6].

As part of these programs, herpetologists observe the tortoise behaviour in order to identify the nests and to retrieve the eggs. It is possible to monitor eggs in their natural environment, however the eggs are often taken to a protection centre. This ensures a lower mortality of the hatchlings during their most vulnerable period [5]. This is repeated every year during the reproduction period. Only during these periods is it possible to observe the females digging activity and to identify the location of the nests. Unfortunately this procedure cannot be applied on a large scale, as it is dependent on the direct observation of the animals. For large-scale applications an automatic system is necessary—one which is able to recognize the behaviour of the animal, and consequently promptly locate the nests.

In this context, our general objective is to design an automatic system called Tortoise@, which aims to recognize digging activity and locate the nests. This is done using a combination of a small device attached to the carapace of wild animals, and an activity recognition system (ARS) based on machine learning methods. This system is based upon, and claims the benefit of, Patent Application Number filed MI2011A002337.

The device should be worn comfortably by the animal, and it should be operational during the entirety of the breeding season (e.g. about two months in Italy for Mediterranean tortoises). Thus the device should be very light in weight, small in size, and energy efficient (i.e. a low computational power and a small memory). The device must be energy efficient because it will need a portable supply of power, i.e. it must be battery powered. In an effort to augment the operational life of the system, the device must be able to recognize the digging activity autonomously. This enables the system to be set up to avoid sending a continuous stream of data, and instead only send the current geographic coordinate when a nest digging event has been identified. According to the need of limited computational and memory capabilities, the ARS must find a good trade-off between accuracy, memory, and computational power.

Another feature of our approach is the use of an accelerometer to detect characteristic movements of the tortoises. Our observation on data collected during the initial experimental campaign show that lateral movements registered by the accelerometer during the excavation of the nest are regular, regardless of the size and species of the tortoise. These characteristics affect only the amplitude of movement, and not its type. The occurrence of a regular pattern, and the presence of the typical data noise, reinforce the decision to use a data-driven, robust, yet simple, pattern recognition approach to automatically identify such patterns.

Keeping in mind these objectives and observations, in summary, our scientific goal is to assess the possibility of introducing an efficient and effective recognition system for the digging activities of tortoises by means of accelerometers data and to support it by an experimental validation from real-data.

The use of neural networks, which have a flexible architecture, allows us to investigate models with low computational and memory requirements. Furthermore, Tortoise@ takes advantage of modular composition of very small neural network models combined with a filter of the model output stream to identify the activity of tortoises. In the paper, both the model and the filter are referred to as an ARS, with name TartaNet. Tortoise@ is part of the biologging research area because of the environmental purpose, and the technology involvement. The biologging field was introduced in [7–9] and described by Bograd et Al. in [10]. Biologging researchers investigate the behaviour of animals in the wild or in semi-free conditions. Ropert et Al. [11] use hall sensors and a logger to study the feeding behaviour in birds and koalas. Baranes et al. [12] and Kays et al. [13] apply biologging techniques to classify animal behaviour based on oyster catchers (*Haematopus ostralegus*) and on toucans, respectively. The processing of animal activity accelerometer data, with the machine learning algorithms, has, with time, become a more accepted approach to monitor the animal behaviours.

Several works in literature present the use of machine learning algorithms for animal activities recognition with different ethological purpose, taking into consideration typically accelerometer data of daily activities, such as feeding, sleeping, and walking, among others. Recently, for example in [14] machine learning algorithms are proposed for behavioural patterns of farm animal (cows) to identify daily activities. A similar approach is used in [15] to store, annotate, and automatically recognize the activity of wild animals. In [16], the machine learning algorithms are proposed as a classification framework for recognizing dog activities. In this concern, the artificial neural networks (ANN) [17] represent a possible approach to provide recognition capabilities to embedded on devices and it is therefore considered in this paper. Focusing on ANN for animal activities recognition, it is to note some recent works. Examples of possible real world applications of this technique are described in [18, 19], and [20], wherein the ANNs are proposed to classify the daily activity of several species.

## 2 Materials and Methods

In the following sub-sections we describe the subjects and the device used for this research, and we present the methods proposed to reach our goal of animal activities recognition.

### 2.1 Ethics statement

The device was gently attached to the carapace with minimal pressure. We used one hundred tortoises in this research and observed their behaviour two to three times a day, for at most one hour. The glue pad used for the tortoises is the one described at the following URL: http://www.uhu.com/en/products/glue-pads.html. The glue pad remained on the animal for a maximum of 3 hours. The procedure is non-invasive, does not cause pain or death of animal, and therefore these studies did not require consultation from the Ethics Committee of the University of Pisa (protocol n. 86/609/CEE). The procedure was carried out in the Protection Centre for Mediterranean Tortoises, in Massa Marittima, Tuscany, Italy. This is a Centre for conservation, ex situ of fauna (CESFA). *The Museum of Natural History*, of The University of Pisa, and *The Unione dei Comuni Montani Colline Metallifere* (UCMCM), are authorities responsible for the centre and for the protection of the inhabiting animals. The owners gave the permission to conduct the procedure inside the centre. Both the UCMCM and *The Museum of Natural History* approved the procedure and the Tortoise@ research conducted by the University of Pisa. In the additional information added to the manuscript, the authorizations are available as Supporting Information files.

### 2.2 Subjects

Our subjects were healthy adult female tortoises from the Protection Centre for Mediterranean Tortoises. In the centre, the tortoises occupied fenced areas divided according to either species or populations of Mediterranean tortoises (Testudo hermanni, Testudo graeca, Testudo marginata in semi-free conditions). The fenced areas of adult tortoises ranged from 180*m*^2^ to 6.25*m*^2^, depending on the number and size of the tortoises. The terrain was hilly and grassy with Mediterranean shrubs. An extensive campaign of data collection was conducted in the field in the June and July of 2012. For our behavioural study, each subject was kept under surveillance throughout the experiments in order to identify its activities.

### 2.3 Hardware equipment and selection of sensors

For data gathering purposes, we used a system comprising two MicaZ devices [21] and a standard PC. A MicaZ is a low-power programmable device with a communication board (with a 2.4 GHz radio subsystem), a MPR2400 antenna, and a radio. The MicaZ radio is based on the IEEE 802.15.4 standard, which guarantees low energy consumption and long-lasting operations [22]. The MicaZ device used as tag attached on the animals is augmented with the MTS310 sensor board which, in turn, has a temperature, a light sensors, and a two-axis accelerometer (the ADXL202). The light and temperature sensors are intended to provide context information, describing the individual’s surroundings. However, these sensors alone cannot provide sufficient information on the tortoise activities. The light and temperature sensors are intended to provide context to primary information, describing the individual’s surroundings, while the accelerometers are used to capture the movements of the animals [20, 23], and [24].

In our experiments we used the following setting for the data collection: one MicaZ (the dispatcher), attached to the animal, was used to record and transmit data concerning the environment, as well as on the movements of the tortoises. The second MicaZ (the collector), which was connected to the PC via USB cable, received the data transmitted by the other MicaZ and provided aggregated data to the PC for storage and off-line analysis. The transmitted data was used to identify the different activities. The dispatchers recorded light levels, temperature, and the accelerometer signals at a frequency of 4Hz (every 250 ms) and stored them in the internal memory of the MicaZ. In fact, this frequency proved afterwards to be excessive, and the sampling could be reduced without impacting the behaviour of the system.

**Fig 1:**
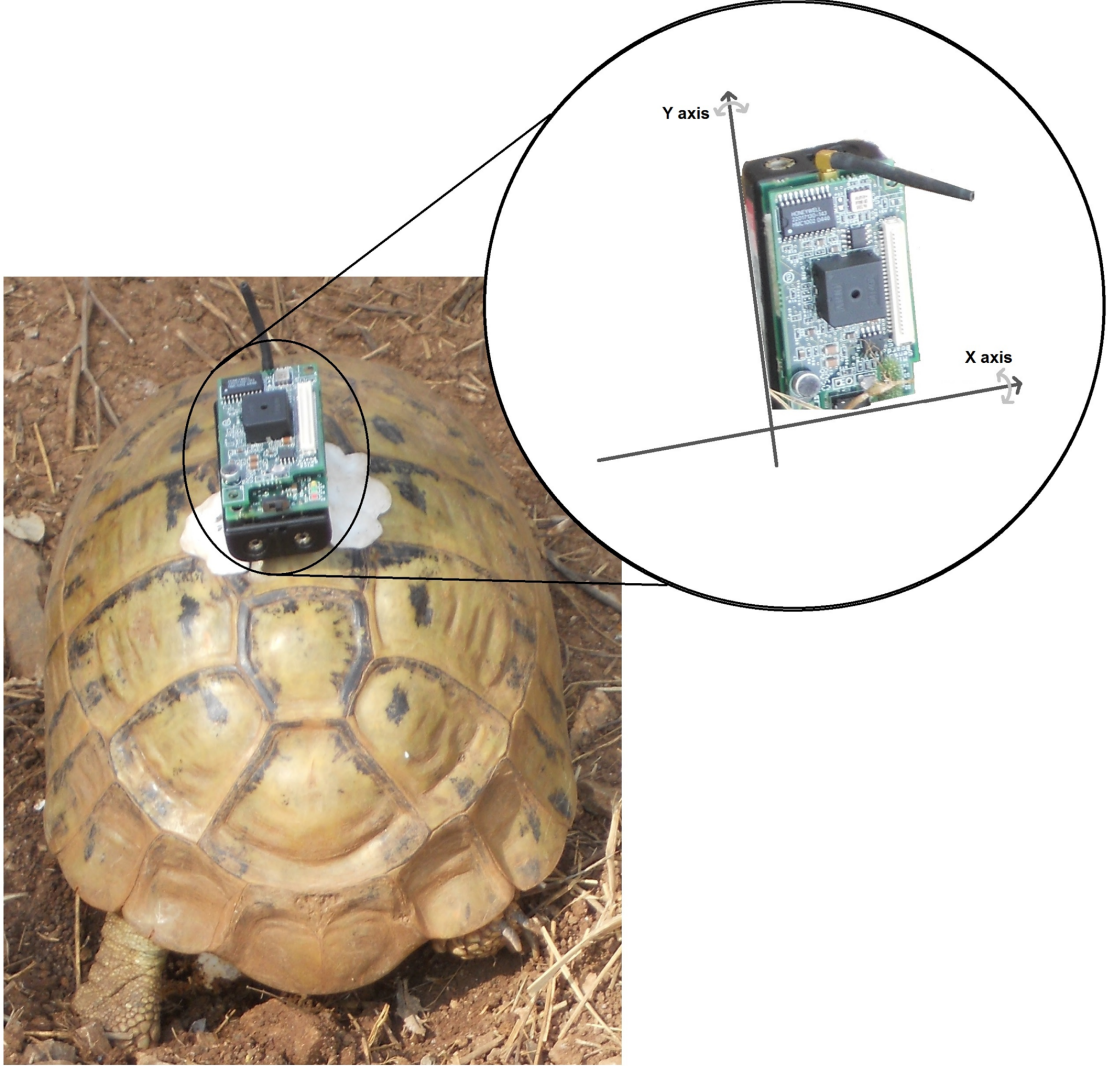
Accelerometer axis. The device is positioned on the carapace with a great care by the supervisor at the beginning of each data collection procedure.

The accelerometer signal was ±2*g*, and described the movements of the carapace along the *x* and *y* axes of the sensor. As shown in Fig 1, the *x* described the movements of carapace along the short side, and the *y* axis described the inclination along the long side of carapace.

**Fig 2:**
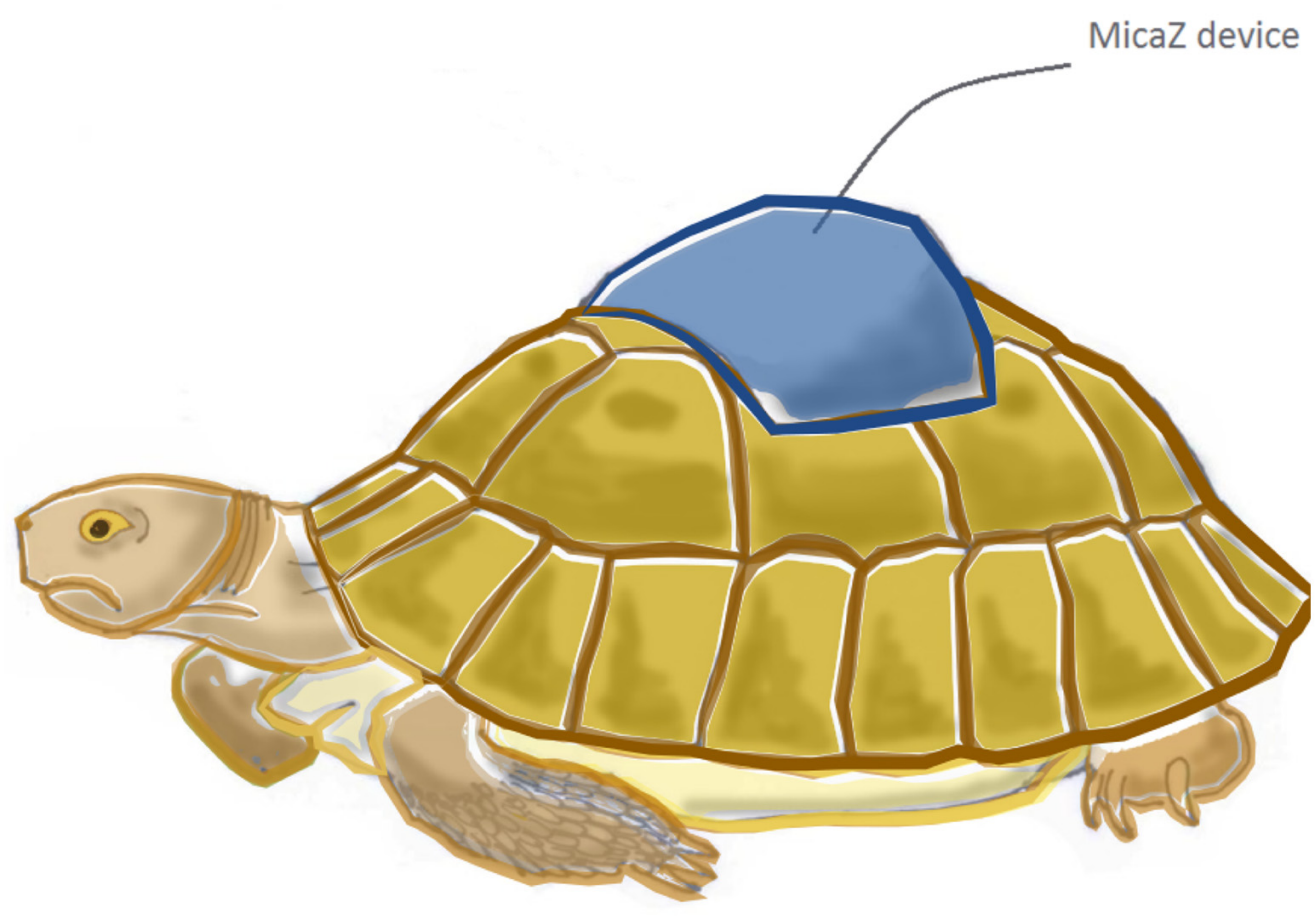
Final device. Detail of the position of device on the tortoise carapace.

For practical use in the wild, the prototype will require a further engineering step to reduce its size and be more comfortable for the animals. The engineered system would need to be unobtrusive and follow the design of the carapace of the tortoises as proposed in the patent and shown in Fig 2.

### 2.4 Data collection protocol

Experiments were performed, with the tortoises, in a semi natural habitat. This environment enabled us to identify the characteristics of the main activities of interest for female tortoises. The data collection protocol was supervised by a human operator who observed the behaviour of the tortoises. Data collection was initiated at the behest of the supervisor, subsequent to the identification of one of three main activities: eating, walking, and digging.

The collection procedure consisted of three phases: *placement, monitoring, and storage*. Throughout these phases, the supervisor recorded and stored each sequence of sensor data in a dataset. Each sequence was labelled with the corresponding activity tag.

This dataset was used in the off-line analysis for training and validating the ARS.

- *Placement*: The supervisor attached the device with a non-toxic and re-usable glue pad onto the carapace between the second and third vertebral plate, without interrupting the activity observed. The location chosen for the device was non-invasive and non-restrictive. The position required consistency in order to guarantee a reasonable homogeneity of the collected data. The device used during the data collection is illustrated in Fig 1.
- *Monitoring*: In this phase, the MicaZ recorded accelerometer data with the given sampling frequency, and stored it in its flash memory. The operator recorded the specific activity being performed by the animal in the tortoise activity diary in order to associate this activity with the sampled data.
- *Storage*: At the end of the activity, the supervisor retrieved the sensor from the carapace, for another recording, and downloaded the signal stored in the flash memory of the mote onto the computer.

Note that it was not possible to establish the duration of each activity in advance. For this reason, a specific time slot was not used during the recording phase for the storyboard of the actions, and the length of each recorded signal was based on the duration of each individual activity, with an upper limit of two hours.

### 2.5 Data Analysis

In the data collection campaign, sequences of sensor data (accelerometers, light and temperature) were collected. Each sequence refers to one nest excavation, walking, or eating activity performed by an individual. The dataset obtained with this dataset is composed by a temporal series of accelerometer raw data.

#### 2.5.1 Pre-processing methods

Pre-processing techniques were used to reduce the noise of the signal and to normalize the values in a uniform range. We also down-sampled the sequences in order to experiment with different sampling rates, for the sake of memory parsimony.

- *Filtering*: in order to filter out noise, we applied a moving average filter, which is effective in the case of non-uniform noise [25]. The moving average filter is a type of Finite Impulse Response (FIR) [26]. This filter operates by averaging on an interval of five points from the original sequence to produce each point in the filtered sequence.
- *Normalization*: the integer values produced by the accelerometers were scaled down by the average of all the observed values—note that this is not a proper normalization. The reason for using this scheme, rather than a more conventional normalization, was to obtain an input stream for the neural network in the form of integer values (instead of float values). This helps a substantial saving in memory, and enables the implementation of the system on memory constrained devices.
- *Down-sampling*: Down-sampling involves reducing the sampling rate of a sequence of data. For our purposes, we adopted the simple mechanism of excluding samples at a given rate from the original sequence. As mentioned before, we used this filter to test the system with different sampling rates, by reducing the number of inputs of the system. The down-sampling is applied on the sequences filtered with the moving average filter.

#### 2.5.2 Input of the neural network

Since the accelerometer has two axes, an accelerometer sequence is in the form of paired sequences: one for each axis.

**Fig 3:**
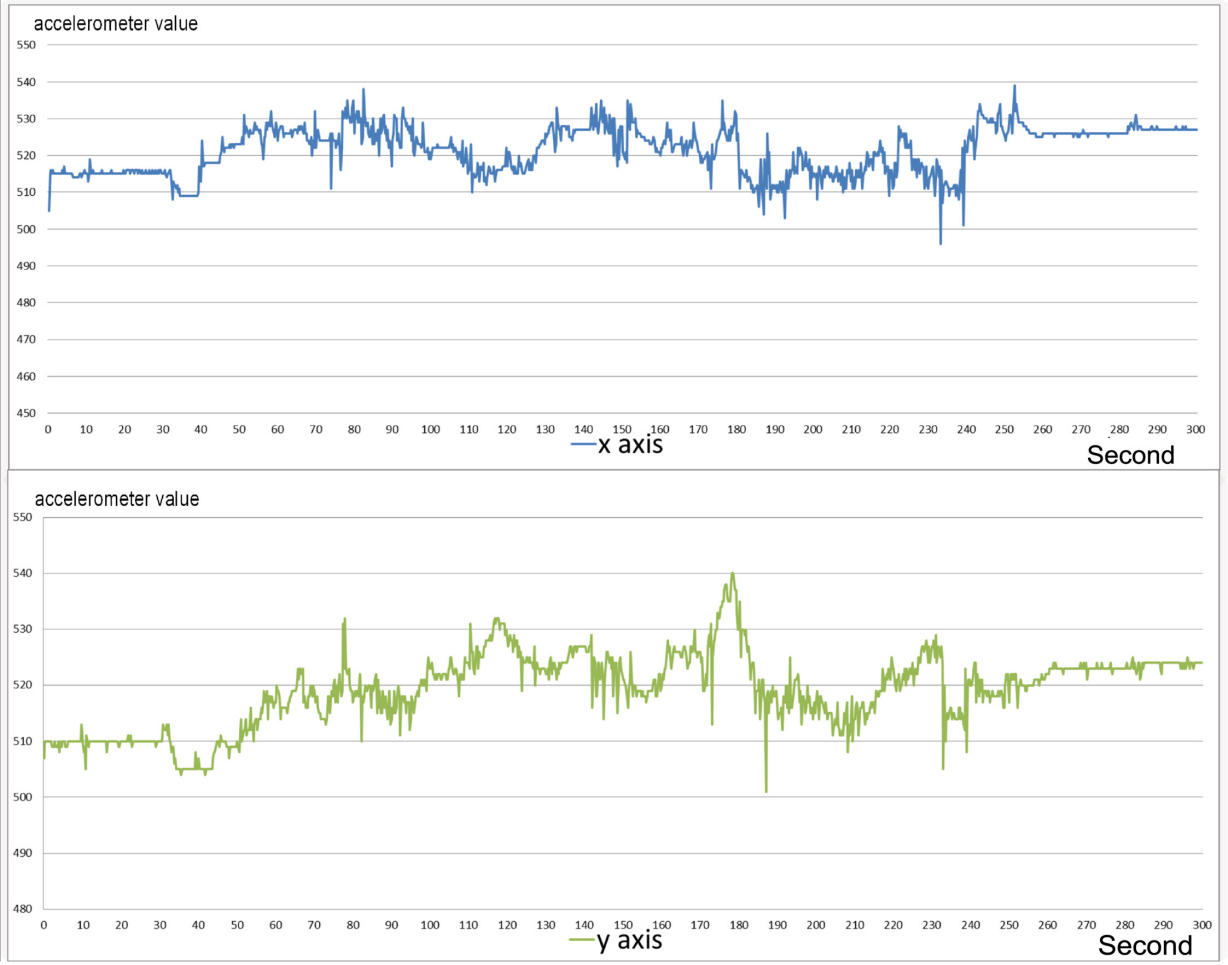
Sequence of a walk phase from the accelerometer. Walking activity, as represented by the blue signal (x axis), is characterized by chaotic oscillation of values, representative of the oscillation of the carapace. A similar behaviour is observed in the green signal (y axis).

**Fig 4:**
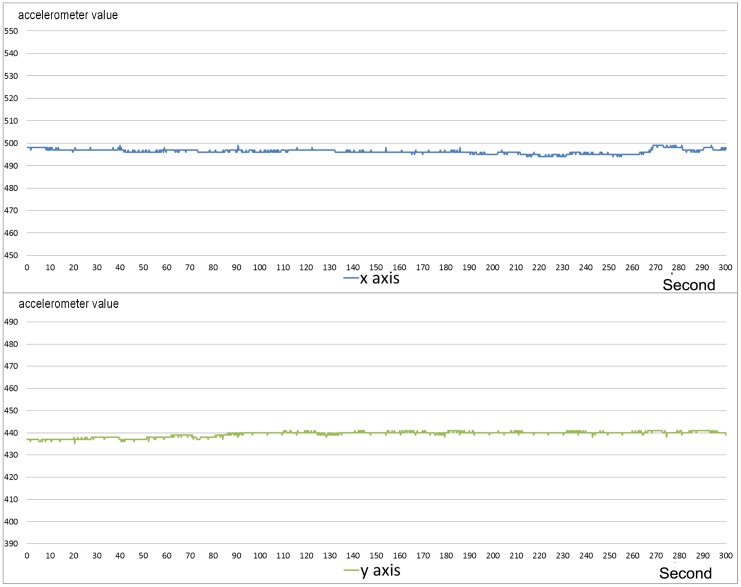
Sequence of a eating phase from the accelerometer. In this phase, the carapace moves only to let the tortoise to reach food outside the reach of its head. Both x and y axes show relatively insignificant variation.

**Fig 5:**
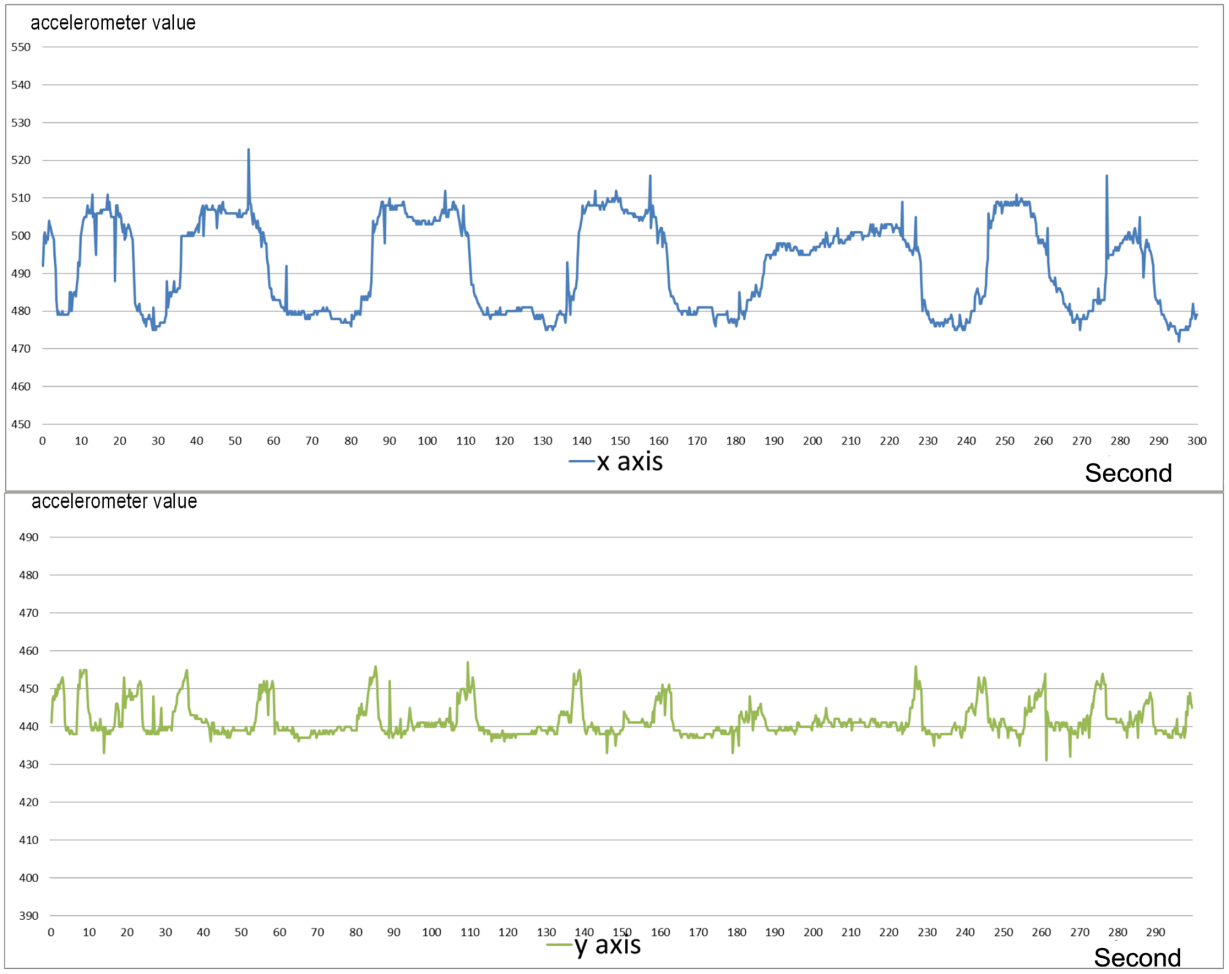
Sequence of a digging phase from the accelerometer. The blue signal (x axis) shows a periodic movement of the carapace along the x axis that resembles a square wave. Each square wave observed on the signal of the x axis corresponds to a peak of the signal of the y axis (the green signal).

Fig 3 shows an example of a walking sequence. In this case the accelerometer sequence is chaotic and hardly predictable. Fig 4 shows a sequence recorded during eating, which is characterized by the complete absence of movement. Fig 5 shows a sample of the digging sequence. The x axis sequence obtained during a digging sequence approximates a periodic square wave. This periodicity is due to the turnover of posterior paws during the excavation.

The accelerometer sequence on the y axis provides negligible information for the discriminative aim, therefore the rest of the paper, is focussed on the analysis of the x axis data, which contributes to reduce the system load for data storage and processing. The database of this study consists of a set of 83 labelled accelerometer sequences. The set is divided into 15 walk sequences, 6 eating sequences, and 62 digging sequences, of which 13 terminated with an egg deposition. The other 49 excavation sequences which were not terminated with a deposition is a result of normal tortoise behaviour in captivity.

The sequences of the accelerometer data are indexed over the time dimension by describing the tortoise activity as a set of successive samples. Each sample corresponds to a quarter of a second related to the frequency of sampling (4 Hz). As mentioned in Section 2.3, to reduce the size of the sequence, we down-sampled it to 1 Hz, with a one second interval (a *time step*).

Each time step is classified by an ARS, which is fed with a window of *n* previous steps (here after, the *input window*). At each time step, we shift the input window by one interval of a quarter of window and obtain a new output value. This particular use of the input windows can be allowed by a neural network model, known as the Input Delay Neural Network (IDNN). This approach is popular in the literature of activity recognition as described in [27].

**Fig 6:**
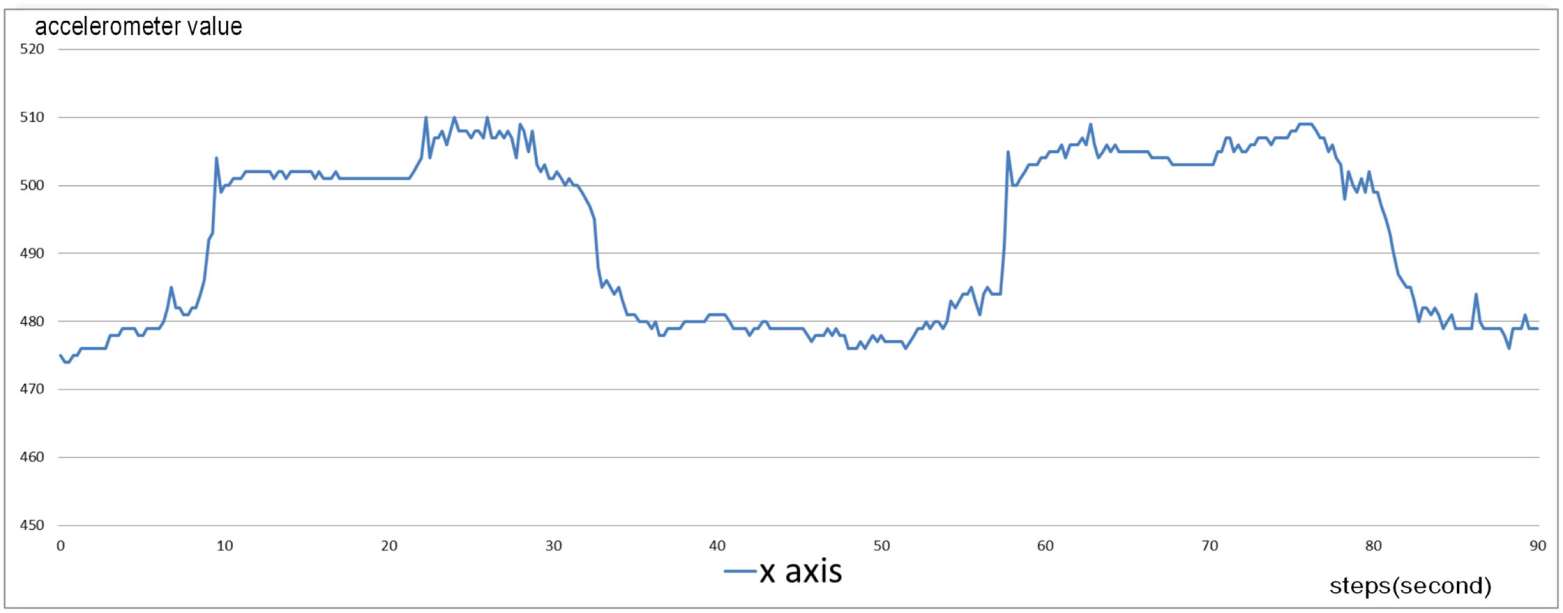
The characteristic pattern extracted from the x axis accelerometer data of a digging sequence. This pattern shows the characteristic periodic movement performed by the tortoise in the digging activities. It is composed of two squared waves which distinguish the excavation sequences. The signal of characteristic pattern is shown in figure with a frequency of 4Hz. The time interval shown in the histogram corresponds to approximately 90 seconds.

Given the main role of the input window, we identified a characteristic pattern of the excavation phase that can be recognized by the neural network. The pattern occurs in a window containing a repetition of the squared wave, as shown in Fig 6, and plays a key role in training the ARS. The period of time chosen for the pattern is the mean duration of two squared waves in the excavation sequences. This corresponds to approximately 90 time steps (90 seconds) in the real data at hand for Mediterranean tortoises. We therefore set the input window to have the same dimension—90 time steps. The dimension of this pattern may vary, depending on the specific species of tortoise under study. In those cases, the size of the window should be changed accordingly in order to include a minimum of two repetitions of squared waves. Note that the variance of frequency was one of the main factors in choosing the pattern dimensions. In our case, to guarantee a generalization of characteristic pattern for Mediterranean tortoises, we took into consideration samples collected by 3 different families of *Testudo*. The great difference between these species allows us to analyse various frequencies of the same type of movement, and then to deal with these variations automatically, through learning, over the different sampled cases in the gathered data set.

The ARS is trained to identify the characteristic pattern and can be utilised to recognize it in long sequences. To find a trade-off between the ARS responsiveness, the memory overhead, and the need for providing enough information to identify the digging activity, we introduce the concept of a sub-sequence extracted by the long sequences, named segment of sequence in the rest of the paper. Taking into account this trade-off, the size of the segment is 5 minutes (300 intervals). Given the initial delay due to the input window (of 90 time steps) and the time interval chosen for the shifting window (one quarter, that is 22 time steps), the neural network provides an output stream of 10 classification values for each segment. Table 1 summarizes the length of the input window, pattern, segment of sequence, and output stream.

**Table 1:** Lengths and descriptions of the input window, pattern, segment of sequence, and output stream.

Input window (buffer)	90 values	input to the NN
Pattern	90 time steps	sub-sequence used to train the neural network
Segment of sequence	300 time steps	accelerometer sequence used for the classification
Output stream	10 values	output values of neural network over the course of a segment

The basic idea beyond this setting is to use these patterns rather than sequences in the training of the neural network to keep a low memory overhead and to improve the performance of the classification prediction by focusing the activity recognition algorithm on the most specific information available about the digging phase.

According to the characteristic pattern, the neural network is trained with samples of 90 seconds labeled with positive and negative classifications. The samples with positive classification are patterns extracted from nest excavation sequences. The samples with negative classification are obtained with patterns sampled by eating and walking sequences.

### 2.6 TartaNet activity recognition system(TartaNet)

The TartaNet activity recognition system (TartaNet: TARTAruga, which means tortoise in Italian, and neural NETwork) integrates a machine learning (ML) model (which is fed with the input windows), and a filter of the model output stream. The machine learning model identifies, within each segment, the windows similar to the characteristic pattern. The TartaNet filter classifies the output stream of machine learning model as a digging or non-digging activity.

#### 2.6.1 Machine learning model of TartaNet

To build the ML model of TartaNet, we focus on the Artificial Neural Network (ANN) in the form of the well-known feedforward ANNs/Multi-Layer Perceptron (MLP) architecture [17]. The robustness and the ability for universal approximation of this method provides the basis for the flexibility of the approach for the approximation of arbitrary classification functions from experimental data, despite not having a theory of the pattern characteristics.

The MLP is a network of computational units organized into one input layer, one or more hidden layers, and an output layer. The possibility to set up different numbers of units allows us to easily investigate different configurations to find a good tradeoff between model complexity and accuracy of the results, which is particularly demanding for this application, as discussed in the previous sections regarding efficiency constraints.

The MLP supervised learning algorithm allows adapting free parameters used to weight the connections of the model, in order to obtain the best approximation of the desired outputs (*targets*). This is typically realized in terms of minimization of an error (or loss) function on the training dataset. In particular, in the classical least-mean-square approach, the error is computed as the square of the difference between the output of the neural network (computed by a Sigmoid Tanh function in our setting) and the target values over all the training samples. For MLP, the backpropagation technique [28] is the most popular among the supervised training algorithms. The backpropagation learning algorithm is based on a gradient descent of the loss function through the weight space in order to reduce the error, allowing tuning the model free-parameters for the training data. Variants including regularization approaches are considered (see Section 3.1). The gradient descent is repeated until the improvement of the error is no longer significant. Once the model is trained, we evaluate the prediction on new sequences of activities.

In order to deal with sequence (of sensor) data by MLP, we focus on the shifting window models. In particular, we consider the Time Delay Neural Network models developed for sequential data (e.g. in [17, 29, 30], and [31]), models which are based on a MLP architecture, and specifically on two models: an Input Delay Neural Network (IDNN) [17], and IDNNs inspired by characteristics of the Convolutional Neural Network (CNN) [32].

**Fig 7:**
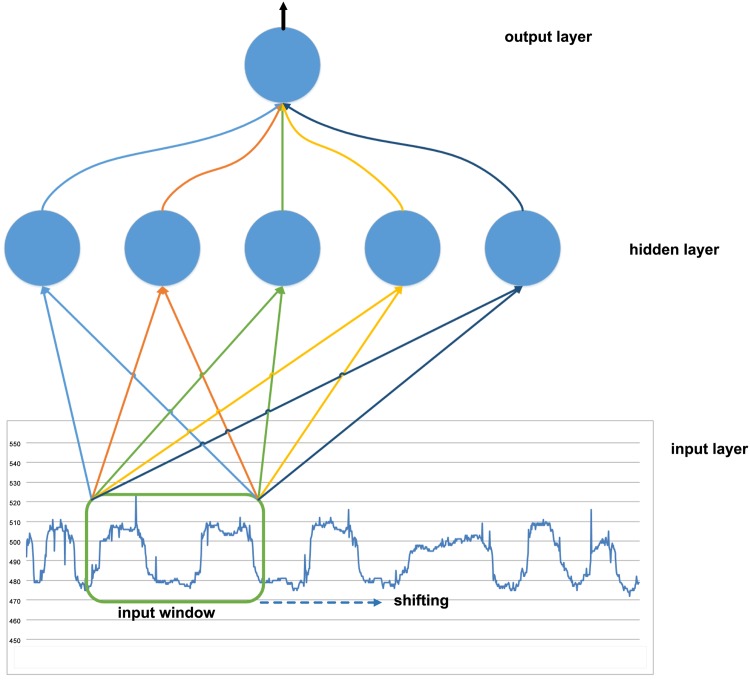
Input delay neural network (IDNN) with an input sequence. An input window of the signal sequence is used to feed each hidden unit. The input window is shifted on the input sequence in order to analyze the entire sequence.

**Input Delay Neural Network**: The dynamic nature of the accelerometer sequences needs a model that can recognize a movement pattern, irrespective of the precise location in time. Thus the neural network needs to represent the relationship between events in time, without a particular temporal alignment, and the Input Delay Neural Network (IDNN) model satisfies this requirement. Fig 7 shows the IDNN model. This model exploits a MLP architecture (all connections are feedforward) with sequential inputs. In particular, in the IDNN model, the inputs to hidden units consist of the outputs of the input units not only during the current time step, but also during previous time steps. As shown in Fig 7, this is implemented using the input window which is shifted on the segment of sequence, which equivalently corresponds to a delay in the units reading the history on the inputs.

The shifting window is highlighted over the segment of sequence, and it feeds the hidden units of the IDNN. Different weights are used for each hidden unit, which in turn feeds the output unit.

The window takes into account the temporal structure of the pattern defined in Section 2.5.2 to train the IDNN. Once trained, the IDNN is used to read the segments of sequences, over time, using the same trained model. In this way the model implements the property of translation invariance [33], identifying the characteristic pattern regardless of its position in the sequence.

Typically, due to the use of different weights for each hidden unit, the IDNN network requires memory occupation for the storage of the weights that can easily exceed the memory available in low-power devices (see details in Section 3.3). This prevents us from using the IDNN with the original architecture in the Tortoise@ application. For this reason we also consider IDNN inspired by the CNN, as discussed in the next section.

**Local receptive fields in input window and weights sharing**: The CNN is a biologically-inspired trainable architecture that learns invariant features from data [32]. The model is introduced in [34, 35] and explained in [36, 37]. Partially inspired by the CNN, we propose to modify the IDNN as described below.

**Fig 8:**
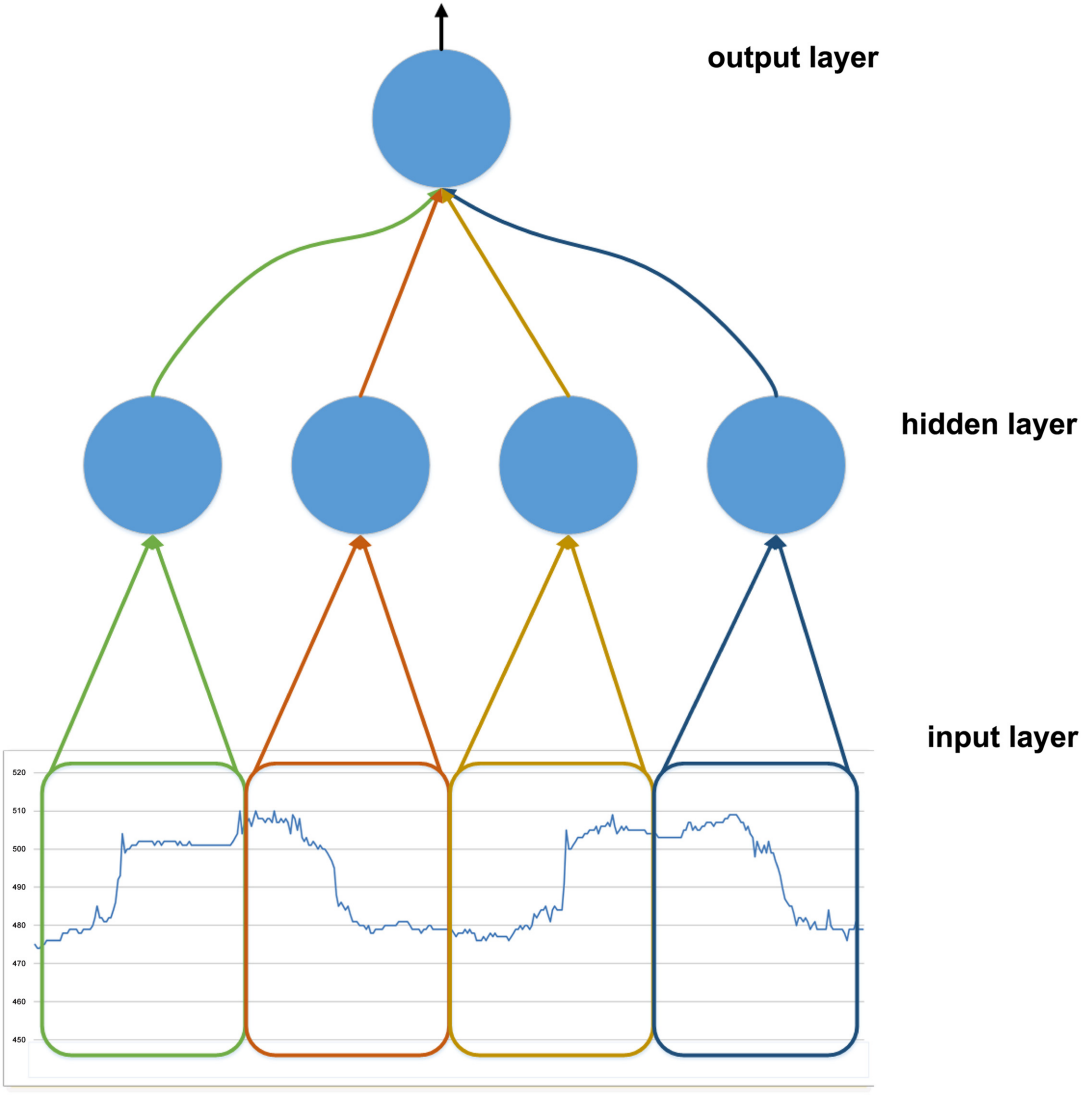
Input delay neural network with local receptive fields (IDNN LRF). The input window is divided in four sub-windows. Each sub-window is a LRF of a hidden unit.

The first variation is to exploit the local receptive fields (LRF) concept from CNN and applying it inside the input window of the IDNN approach. The main idea of LRF is to connect units in a layer to receive input from a set of units in a small neighbourhood of the previous layer. Each hidden neuron scans the input using their local receptive field. A unit with local receptive field obtains an inventory of features to represent the characteristics of the sub-window (as described by Lang et al. in [30]), without being burdened by an excessive number of free parameters and reducing the memory space [17, 38]. Specifically, we identify four input sub-patterns in the characteristic pattern (shown in Fig 6). Two of these sub-patterns identify the ascent phases of the squared waves and the other two identify the descent phases, as shown in Fig 8.

According to the split of the characteristic pattern, the sub-patterns are then mapped on the input window of the neural network. We then divide the input window into four sub-windows (each sub-windows is formed by 22 inputs). As a result of this specialization (namely IDNN LRF), the hidden layer is composed of four hidden units, each of which is connected to a sub-window by independent weights, and consequently is focused on a specific phase of the characteristic pattern. As a result, each hidden unit takes 22 inputs and therefore the model requires the storage of 22 weights for each of the four hidden units. This reduces the memory occupation for the weights of the classical IDNN, which require 90 weights for each hidden unit.

**Fig 9:**
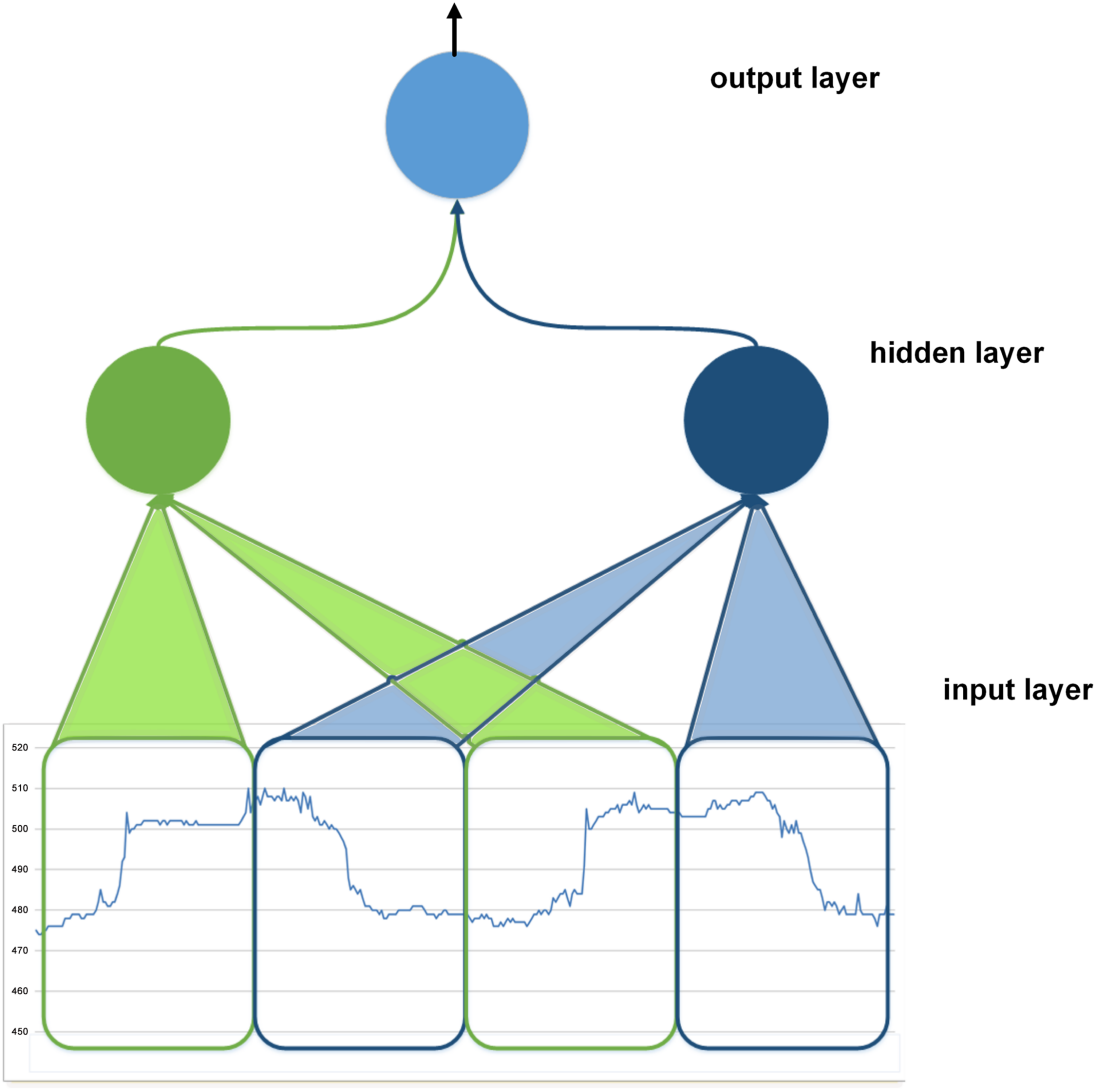
Input delay neural network with LRF and weight sharing (IDNN LRF WS). The input window relates the four sub-windows identified for IDNN LRF. With characteristic pattern divided in sub-windows, both the first and the third sub-windows are in an ascent phase of the squared signal, whilst the second and the fourth sub-windows are in the descent phase. The colours of the connections highlight the sharing of weights between hidden units due to the match between sub-windows. Weights are shared between the two sub-windows that make up each phase: i.e. each unit uses the same set of weights for both the sub-windows of the ascending and descending phase (we identify this model as IDNN LRF WS).

The second idea inspired by CNN is weight sharing (WS) among LRF hidden units, which constrains the weight vectors of some hidden units to be equal. This further reduces the number of free parameters from the large amount of units sharing the same weight vector and obtains a certain level of shift invariance (the detection of features regardless of their position). We implement weight sharing between the sub-windows that make up the characteristic pattern, i.e. two sub-windows of ascent and two sub windows of descent. This reshapes the IDNN structure by reducing it to two hidden units: one for each phase, as shown in Fig 9.

In this way the memory occupation accounts for 22 weights of two hidden units each (half of the memory occupation obtained with the sole LRF method). Table 2 shows the reduction of input weight vector introducing sub-windows.

**Table 2:** The column # subwindow lists the sub-division of the input window related to the different models. The column # weights per hidden unit lists the number of weights necessary for each hidden unit (including one more for the bias) related to the different models according the dimension of their input field.

Model	# sub-windows	# weights per hidden unit
**IDNN**	no sub-window	91
**IDNN LRF**	4	23
**IDNN LRF + WS**	2	23

#### 2.6.2 TartaNet filter for neural network output stream

The models described in the previous section classify each input window that makes up a segment of sequence. Hence, for each segment, each model provides an output stream made up of classification values defined in the range [−1,+1], where a positive output value corresponds to an input window similar to the characteristic pattern.

**Fig 10:**
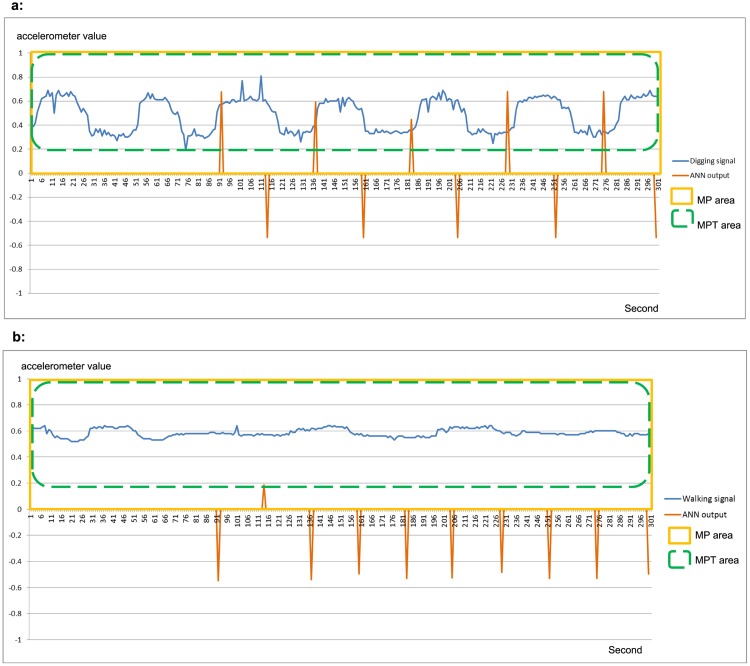
Output of IDNN LRF. **a)** The light blue signal corresponds to the movement of carapace collected during a digging phase. The orange signal is the response of the IDNN LRF to each (shifting) input window. The IDNN LRF outputs peaks when it finds windows with high similarity to the characteristic pattern. **b)** The blue signal refers to non-digging (walking) movements. The orange signal (the IDNN LRF output) has negative values with one small positive pick due to a window similar to the characteristic pattern by chance. In both graphs the yellow rectangle comprises values used for MP filter, and the green rectangle comprises values used for MPT filter.

Fig 10a shows an example of output stream of IDNN LRF obtained with a segment of digging sequence. In this case many output values are in the range [0,+1]. Fig 10b shows the output stream of the same neural network with a segment of walking sequence. In this case, most of the output values are negative since the input windows do not correspond to the characteristic pattern.

According to the description above, we use an output filter to classify the output stream of the neural network as positive or negative over a segment. The TartaNet filter exploits three features computed on the ANN output stream:

- *NP*(Number Positive): Number of positive values.
- *MP*(Mean Positive): Mean value of all positive values compared to all the output values: *sum of positive values / output stream length*.
- *MPT*(Mean Positive Threshold): Mean value of the positive values above a threshold compared to all the output: *sum of the positive values above τ* / *output stream length*.

Where:

- The threshold *τ* was computed as mean of output positive values obtained with a validation set. The value obtained for the cases discussed in Fig 10 is 0.2.
- The TartaNet filter evaluates each feature individually against a *feature bound*, to assess it as a positive or negative response through a threshold.

**Fig 11:**
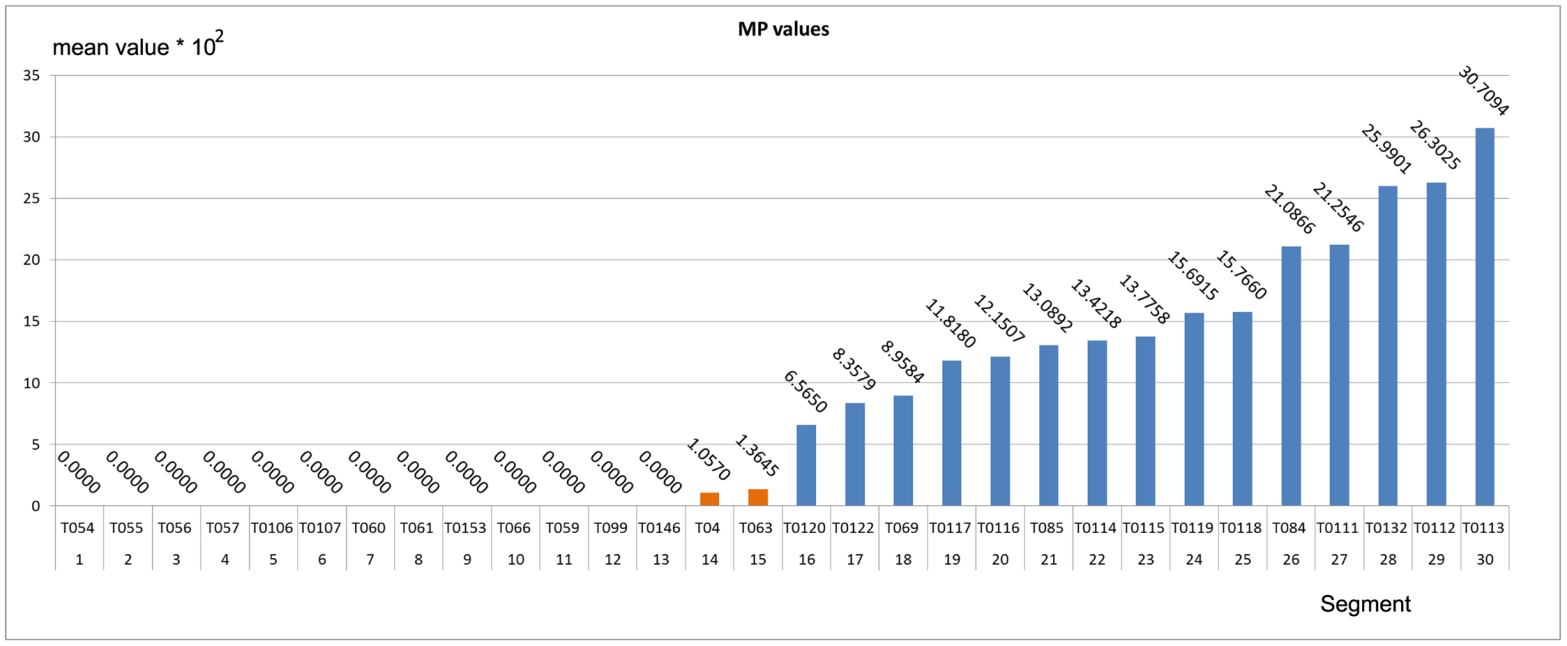
Histogram of the *MP* values of IDNN LRF output stream. Each column relates to the average of the positives values in the corresponding segment. The blue columns are the mean values obtained with digging segments whereas the orange (or zero) ones are the mean values of non-digging segments.

**Fig 12:**
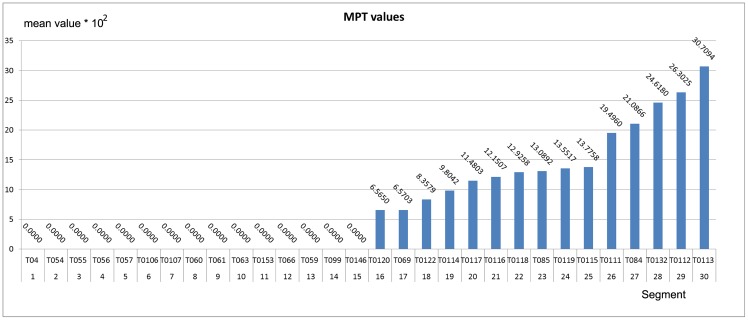
Histogram of the *MPT* values of IDNN LRF output stream. Each column relates to the average of the positives values above a threshold *τ* in the corresponding segment. The blue columns are the mean values obtained with digging segments whilst the orange(or zero) ones are the mean values of non-digging segments.

The bounds of *MP* and *MPT* are useful to distinguish the output stream of one digging sequence from one obtained with a walking sequence. The bounds of MP and MPT are identified by observing the results on a separate dataset, the validation set used in the model selection, explicated in Section 3.1. These results are shown in the histograms in Figs 11 and 12. The histograms show the *MP* and *MPT* values obtained during the validation phase using fifteen negative segments (non digging sequences) and fifteen positive segments (digging sequences). Note in Fig 11 that the *MP* values of the output streams have a gap between the higher *MP* value obtained with the negative segments and the lower *MP* value obtained with the positive segments, that is between values for segments 15 and 16 in this case. Taking this gap into account, we set the threshold for the the *MP* using the mean value between the two bounds. Using the same approach, we found the bounds for *MPT* in Fig 12.

The value of NP identifies how many positive values are identified in the output stream. The use of NP in the filter enables us to also identify output streams with a few positive responses (which may have a low score over the average values used by *MP* and *MPT*) i.e. even cases in which the segment include few neat positive patterns can be recognized as digging activity. We observed that, with the digging sequences, at least a quarter of values are positive and above the selected *τ*, suggesting a threshold of 2 positive values. The filter then classifies the segment of sequence as positive if and only if at least two features are positive (i.e. above their threshold).

The TartaNet filter provides the classification of the neural network outputs, obtaining a final result to classify the accelerometer data. It is worth noting that the reason for using a modular design combining an NN and an output filter applied to a segment of the sequences lies in the requirement for efficiency, which underlies the aim of our approach. In particular, it allows us to simplify the model and reduce the memory request for the ARS. Specifically, the use of the output filter allows the reduction of the complexity of the NN model (up to only 2/4 hidden units), and to avoid the need of a complex output layer by moving part of the recognition process to the filter. Furthermore, the introduction of the segment of sequence (Section 2.5) allows us to reduce the ARS memory overhead.

Finally, this design makes it possible to produce a result for each input segment (every 300 seconds), thus giving the opportunity to repeat the classification process several times on disjointed segments (the entire digging sequence may last up to 2 hours). As a result we can reach high global classification accuracies without requiring the learning over entire sequences and therefore without additional memory costs in the implementation of the model. In other words, the reliability of the classification (that can be confirmed over disjointed segments) is not, in this way, at the cost of the dimension of the model architecture.

## 3 Results and Discussion

We performed the evaluation of the proposed models, IDNN, IDNN LRF, and IDNN LRF WS, for TartaNet with the aim to find a good trade-off between their performance and applicability.

### 3.1 Model validation

For models training, validation and evaluation, we split the segment dataset into four parts:

- ANN training set: these segments are used to generate the positive and negative patterns to train the neural networks. The resultant training set is composed of 134 balanced input patterns of 90 seconds each (67 positive and 67 negative).
- ANN validation set: these segments are used to generate the validation set of patterns to configure the neural networks (in our case 10 positive and 10 negative patterns of 90 seconds).
- Output filter validation set: these segments are applied to configure the thresholds of the output filters. This dataset is composed of 30 segments of five minutes (15 positive and 15 negative), which are disjoint from the training and validation sets described above.
- Test set: the segments of this external dataset are used to check the overall performance of the final TartaNet system on a set disjoint from the training and the validation sets. This final evaluation is useful to estimate future behvaiour of the whole system. The test set is composed of 56 segments of five minutes each (28 positive and 28 negative).

Each model is trained with the ANN training set, composed in equal parts of positive and negative patterns. The ANN validation set is used to select the best configuaration (model selection) on the basis of the classification accuracy with the distinct patterns. Then the output filter is configured on its validation set to evaluate its behavior for the entire period of the segments. After the validation phases, we evaluate the performance of the selected models with the test set (using new data, unseen in the training and validation phase).

Note that the corpus of ANN training and validation sets accounts for approximately 30% of the entire data set, while the rest of the data is used for output filter validaton and test set (the latter is approximately 42% of the data).

For the ANN training phase, we assume two different initialisation ranges for the initial weights. The weights connections between the input layer and hidden layer are initialized within the range of [−0.00001,0.00001]. The weights connections between the hidden layer and the output layer are initialized within the range of [−0.01,0.01]. The number of units for the input layer is fixed to 90, according to the dimension of the pattern, for all the ANN models.

The validation phases of the ANN allow us to configure the *hyperparameters* values, specifically the number of hidden units, learning rate, momentum, and a parameter of weight decay [17]. The number of hidden units determines the size of the neural network and its learning capability. This is a hyperparameter for the IDNN model, whereas for the other two models there is a fixed structure related to sub-windows. For IDNN we considered values in the range [1, 10]. In particular, we assessed IDNN as 5 units, which is the best trade-off for this model. The learning rate controls the size of the change of weights during the iterations of the learning phase. The momentum is used to stabilize the network convergence. We explored the learning rate and the momentum in range of [0.0001,0.1] and [0,0.1], respectively. To include regularization in the ANN learning, which is useful to control the complexity of the model and hence to improve the generalization capability of models, we use a *weight decay* approach. This corresponds to the addition of a penalty term for complexity (based on the weight vector space norm) to the square error in the loss function used in the model training phase [17] [39]. In particular, this penalty term controls the weights magnitude, specifically the weight matrix squared L2 norm, i.e. the sum of squares of weights values, penalizing models with extreme parameter values. We explored the weight-decay hyperparameter in the range [0,0.001].

### 3.2 Performance analysis

The performance analysis asseses the classification accuracy measuered in terms of percentages related to the size of the sets and the relative confusion matrix for each model.

The performance measurements of the three models take into account the averages of errors computed on five different initializations of their weights for the ANN.

The accuracy obtained with each model is shown in Table 3. For the sake of completeness, the table reports the statistics on the configuration phase on ANN training of patterns (column labeled Training ANN), showing that all the models achieved more than 90% accuracy. Test results correspond to the evaluation on external data for the whole of TartaNet. The table does not report the validation accuracy of the output filter as it was 100% for each model, as per the results from Subsection 2.6.2.

**Table 3:** Classification accuracy of the models.

Model	Training(ANN) (standard deviation)	Test (standard deviation)
**IDNN**	92.59% (± 0.003)	96.24% (± 1.68)
**IDNN LRF**	90.74% (± 0.015)	95.51% (± 0.68)
**IDNN LRF WS**	90.74% (± 0.006)	94.34% (± 0.84)

The IDNN model achieves an accuracy of 96% (with a standard deviation of approximately 1.7 among different model initilizations) in the test phase with 4% of error due to misclassified segments showing as false positives, as shown in the confusion matrix Table 4.

**Table 4:** Confusion matrix of IDNN on the test set.

Real / Predicted	Digging	Non-digging
**Digging**	28	0
**Non-digging**	2	26

With the IDNN LRF model we experience slightly inferior performance (95.5%), but it is worth mentioning that the memory requirements are less than IDNN model (as we show in the section below). The analysis of errors shown in the confusion matrix (Table 5) shows 2 false positives, and 1 false negative.

**Table 5:** Confusion matrix of IDNN LRF on the test set.

Real / Predicted	Digging	Non-digging
**Digging**	27	1
**Non-digging**	2	26

The IDNN LRF WS model has a different behavior due to the high specialization of the hidden units. The 7% of failed classifications are mainly false positivies as in the other two models (as shown in Table 6). The IDNN LRF WS accuracy (94%) is slightly below the other models.

**Table 6:** Confusion matrix of IDNN LRF WS on the test set.

Real / Predicted	Digging	Non-digging
**Digging**	27	1
*Non-digging*	3	25

As explained in Section 2.6, the classification on segments (300 seconds) can be repeated several times (as digging needs a long period of continuous activity, up to a couple of hours). Because of this we can increase the accuracy of the global Tortoise@ system in recognizing the digging activity and virtually completely avoid the few false positives observed in the results. The few false positive classifications (i.e. false digging) shown in the confusion matrices in all the proposed models are obtained with walking sequences. Considering the IDNN LRF model and the two false positives in Table 6 (segments T01 and T03 in the provided data set), we tested the repetition of the classification after 10 minutes (two times the length of a segment) that resulted in a non-digging outcome (segments T04 and T05 from the same walking sequence that are classified as negative by the model) allowing us to avoid a Tortoise@ false identification of the nesting phase. The confusion matrix in (Table 5) also shows a false negative in one segment: T098. This misclassification of a single segment does not avoid the possibility of the recognition of the sequence as a digging sequence. Indeed, the rest of the sequences are composed of positive patterns which can activate in any other instance over the course of nesting the Tortoise@ identification of the nesting phase.

### 3.3 Applicability analysis

In this section we evaluate the three models according to their applicability in low-power devices such as the one used in our data collection. The following discussion holds true for more general cases, including the use of different devices, as it is related to efficiency, which is a central aim for this study, as explained in the introduction. The main concern is the memory occupation of each model, since these devices have a small memory (typically a few kilobytes). We evaluate the memory occupation of the neural network by considering the integer representation of the inputs and the float representation of the weights.

Each of the three models need 90 integer values for the representation of the input window. For the IDNN model, 90 float weights are needed for each hidden unit, and five float weights for the output unit (including one more per each unit for the bias). The IDNN LRF and IDNN LRF WS models, which use sub windows, need 22 float weights for each hidden unit. Note that IDNN LRF has four hidden units, whereas IDNN LRF WS has two hidden units, and the weights for the output unit are four and two, respectively. Table 7 shows the memory footprint of the three models. IDNN has the highest memory footprint. IDNN LRF WS has a very low memory footprint which, however, penalizes its classification performance, while IDNN LRF has a slightly larger memory footprint than IDNN LRF WS, but it offers the best compromise between memory footprint and classification performance.

**Table 7:** Memory footprint. Each row in table relates bytes needed to memorize the input weight vectors of hidden units and output units.

Model	Memory footprint
**IDNN**	1844 byte
**IDNN LRF**	388 byte
**IDNN LRF + WS**	196 byte

## 4 The Tortoise@ System

TartaNet is part of Tortoise@, which is an autonomous system for large scale applications aimed at identifying tortoise nests and rescuing eggs. Tortoise@ is a biologging system preliminary presented in [40] and [41], which enables an autonomous and assistive observation of tortoise digging behavior. It is a custom-made system based on sensors used to monitor movement, and to locate nests. The system is organized into four steps:

- *Environment monitoring*: monitors the environment (light and temperature) to identify the suitable conditions for the deposit eggs. This provides useful contextual information [42].
- *Movement monitoring*: data acquired by the accelerometer sensor are analyzed in order to recognize the digging movements.
- *Extended movement monitoring*: at regular intervals and for a defined short period of time, this phase is repeated to confirm the detection of digging activities, and thus to improve the overall accuracy of Tortoise@, allowing us to set up a reliable system for digging recognition.
- *Data communication*: sends geographic coordinates to a remote user through a base station.

**Fig 13:**
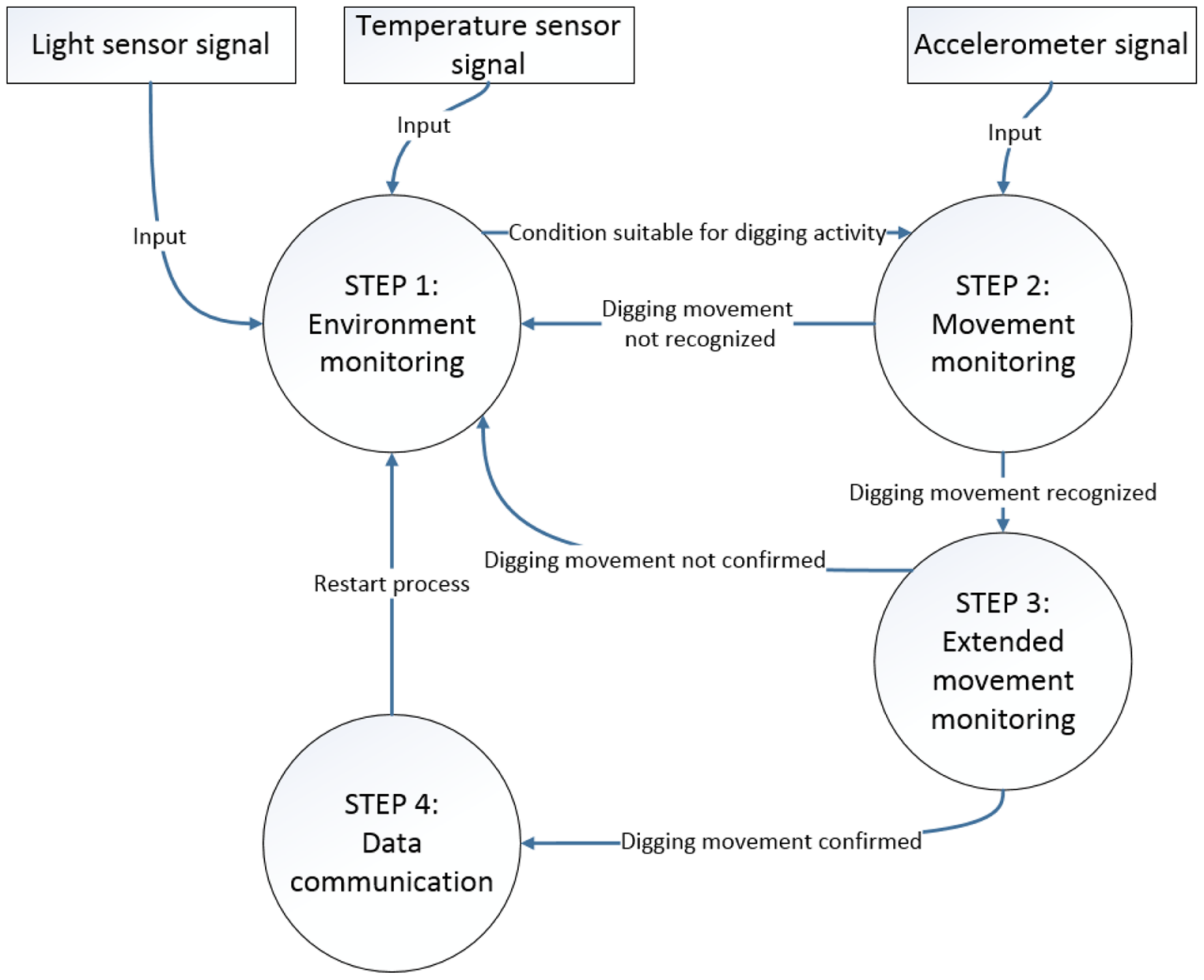
A scheme of the Tortoise@ system. The three input signals (light, temperature and accelerometer), the four steps (in the circular boxes) and the transitions between steps (labelled lines).

The steps of Movement monitoring and Extended movement monitoring are implemented by the TartaNet activity recognition system. The four steps and the transition rules between steps are described in Fig 13. All these steps are implemented within a single device, using local processing for Tartnet to limit communications with the remote user only to the notification of the position of the identified nests. Note that this design meets the requirement of energy efficiency. The analysis of environmental conditions performed in step 1 limits the activation of the neural network implemented in step 2 only to the cases in which the environmental conditions are suitable for nesting. This also limits the recording of the acceleration data stream, which occurs only when step 2 is active and lasts for 5 minutes. When step 2 identifies a digging activity, it then activates step 3 to extend the recognition of the activity in successive time slots (see Section 2.6).

This helps improve the overall reliability of the classifier. If the digging activity is confirmed by step 3 then the system promptly sends the information about the position of the nest to a remote user in step 4. This local processing avoids the burden of having a continuous transmission of data for remote processing, thus granting a longer operational lifetime to the device.

In terms of ethological research the Tortoise@ system provides the opportunity to make automatic an important procedure to assist tortoise populations. The high level of accuracy of the Tortoise@ system makes it particularly suitable for protection programs. The collection of eggs from the wild, the raising of hatchlings in captivity and the return of them to the wild when they are big enough to have a high probability of surviving, is an experimental methodology in conservation management. Examples of this methodology applied to birds and tortoises are reported in [43–46].

The system also identifies information on suitable environmental conditions for nesting and the behavioral characteristics of tortoises during the digging phase. It represents a new possibility for herpetologists to monitor and assist endangered tortoise populations.

## Conclusions

This work was inspired by a concrete applicative problem concerning the protection of tortoises, and, in particular, by the need of biologging devices that may localize tortoises during the escavation of their nest to enable protection programs aiming at retrieving the eggs before the hatching and at protecting the hatchlings in their first year of life.

Although simple in principle, the strong applicative constraints concerning the small size of the device and of its batteries, and the long operational time (the nesting period may last a few months), require innovative and very efficient activity recognition mechanisms that may fit a class of low-power and low-memory devices.

With this requirement in mind, we addressed the scientific goal of finding efficient solutions for the recognition of the digging activities of tortoises, leveraging on data produced by simple sensors like accelerometers.

Our findings, based on real-data gathered in an experimental data collection campaign, show that one dimensional accelerometer (appropriately oriented) is sufficient to catch the characteristic pattern of movement executed by the tortoises during digging, that this pattern is quite short (fits well in 90 samples of accelerometer for the species of tortoises of interest in this study) and that this pattern can be recognized with high accuracy by relying only on few neurons ANN based on the IDNN with Local Receptive Fields model.

This result exploits the modular organization of the proposed activity recognition method (called TartaNet), which combines a very small ANN and a simple output filter. In particular, this design finds a tradeoff between the accuracy and size of the ANN, while the output filter leverages on a few repetitions of the pattern recognition process to achieve a very high overall accuracy of the method.

The assessment of the scientific goal was achieved in a constructive way providing an effective and efficient model specialized for the digging recognition and supported by the experimental results on real-data for the Mediterranean tortoises.

The promising results obtained with TartaNet will enable us to develop a low cost prototype with reduced size, weight and battery pack that can be used on wild tortoises in a natural environment with negligible invasiveness and on large-scale.

In terms of models development, in our future work we will investigate the use of the convolutional neural network model by including further hidden (sub-sampling) layers in the neural network. In this way, the classification procedure and the recognition algorithm would be integrated together. At the cost of increasing of memory occupation, we would expect to further reduce the sensitivity of the output to shifts and distortions trough the sub-sampling of such approach and to achieve a full automatic tuning of the classification procedure.

Future interesting endeavors regard the possible comparison with alternative approaches such as those based on Fourier transform.

## Supporting Information

S1 File Italian law about the use of animals for experimentation. (PDF)

S2 File Authorization of the museum of natural history. (PDF)

S3 File Authorization of Regional office. (PDF)

S4 File Authorization of UCMCM. (PDF)

S1 Dataset Dataset used for experimentation. (ZIP)
