# Supplementary material for: Localizing Tortoise Nests by Neural Networks
Source: PLoS One. 2016 Mar 17;11(3):e0151168. doi: 10.1371/journal.pone.0151168 (PMC4795789; doi:10.1371/journal.pone.0151168)
Supplement: S1 File — (PDF) [file pone.0151168.s001.pdf]

DIRETTIVA DEL CONSIGLIO
del 24 novembre 1986 concernente il ravvicinamento delle disposizioni legislative, regolamentari e amministrative degli Stati membri relative alla protezione degli animali utilizzati a fini sperimentali o ad altri fini scientifici (86/609/CEE)

IL CONSIGLIO DELLE COMUNITÀ EUROPEE,
visto il trattato che istituisce la Comunità economica europea, in particolare l'articolo 100,
vista la proposta della Commissione (1),
visto il parere del Parlamento europeo (2),
visto il parere del Comitato economico e sociale (3),
considerando che tra le legislazioni nazionali attualmente in vigore sulla protezione degli animali utilizzati a taluni fini sperimentali esistono disparità che possono influire sul funzionamento del mercato comune;
considerando che, per eliminare tali disparità, occorre armonizzare le legislazioni degli Stati membri; che tale armonizzazione dovrebbe garantire che il numero degli animali utilizzati a fini sperimentali o ad altri fini scientifici sia ridotto ad un minimo; che tali animali vengano adeguatamente accuditi e che non vengano loro inflitti inutilmente dolori, sofferenze, angoscia o danni durevoli e garantire altresì che, laddove siano inevitabili, questi ultimi vengano limitati al minimo;
considerando in particolare che si dovrebbero evitare inutili ripetizioni di esperimenti,
(1) GU n. C 351 del 31. 12. 1985, pag. 16.
(2) GU n. C 255 del 13. 10. 1986, pag. 250.
(3) GU n. C 207 del 18. 8. 1986, pag. 3.
HA ADOTTATO LA PRESENTE DIRETTIVA:

Articolo 1

Scopo della presente direttiva è il ravvicinamento delle disposizioni legislative, regolamentari e amministrative applicabili negli Stati membri in materia di protezione degli animali utilizzati a fini sperimentali o ad altri fini scientifici, in modo da evitare che l'instaurazione e il funzionamento del mercato comune vengano compromessi, in particolare attraverso distorsioni di concorrenza od ostacoli agli scambi.

Articolo 2

Ai sensi della presente direttiva si intende per:
a) «animale», non altrimenti specificato: qualsiasi vertebrato vivo non umano, ivi comprese le forme larvali autonome e/o capaci di riprodursi, ad esclusione di altre forme fetali o embrionali;
b) «animali da esperimento»: ogni animale utilizzato o da utilizzare in esperimenti;
c) «animali da allevamento»: animali allevati appositamente per essere impiegati in esperimenti in stabilimenti approvati dalle autorità o registrati presso queste ultime;
d) «esperimento»: l'impiego di un animale a fini sperimentali o altri fini scientifici che possano causare dolore, sofferenza, angoscia o danni durevoli, compresa qualsiasi azione che intenda o possa determinare la nascita di un animale in queste condizioni, ma esclusi i metodi meno

dolorosi di uccisione o di marcatura di un animale accettati dalla prassi moderna (cioè, i metodi «umanitari»); un esperimento comincia quando un animale è preparato per la prima volta ai fini dell'esperimento e
termina quando non occorrono ulteriori osservazioni per
l'esperimento in corso; l'eliminazione del dolore, della sofferenza, dell'angoscia o dei danni durevoli, grazie alla corretta applicazione di un anestetico, di un analgesico o di altri metodi, non pone l'utilizzazione di un animale al di fuori dell'ambito di questa definizione. Sono escluse le pratiche agricole o cliniche veterinarie non sperimentali;
e) «autorità»: l'autorità o le autorità designata(e) da ciascuno Stato membro quale responsabile del controllo degli esperimenti contemplati dalla presente direttiva;
f) «persona competente»: chiunque sia considerato da uno Stato membro competente a svolgere le funzioni descritte nella presente direttiva;
g) «stabilimento»:qualsiasi impianto, edificio, gruppo di edifici o altri locali; esso può comprendere anche un luogo non completamente chiuso o coperto e strutture mobili;
h) «stabilimento di allevamento»: qualsiasi stabilimento in cui gli animali vengono allevati allo scopo di essere successivamente utilizzati in esperimenti;
i) «stabilimento fornitore»: qualsiasi stabilimento diverso da quello di allevamento, che fornisce animali destinati ad essere utilizzati in esperimenti;
j) «stabilimento utilizzatore»: qualsiasi stabilimento in cui gli animali vengono utilizzati in esperimenti;
k) «adeguatamente anestetizzato»: privato della sensibilità mediante metodi di anestesia (locale oppure generale) almeno altrettanto efficaci di quelli applicati nella prassi veterinaria;
l) «uccisione con metodi umanitari»: l'uccisione di un animale in condizioni che comportino, secondo la specie, la minore sofferenza fisica e psicologica;

Articolo 3

La presente direttiva si applica all'utilizzazione degli animali in esperimenti eseguiti per uno o più dei seguenti fini:
a) lo sviluppo, la produzione e le prove di qualità, di efficacia e di innocuità dei preparati farmaceutici, degli alimenti e di altre sostanze o prodotti:
ii) per la profilassi, la diagnosi o la cura di malattie, di cattivi stati di salute o di altre anomalie, o dei loro effetti, sull'uomo, sugli animali o sulle piante;
ii) per la valutazione, la rilevazione, il controllo o le modificazioni delle condizioni fisiologiche nell'uomo, negli animali o nelle piante;
b) la protezione dell'ambiente naturale, nell'interesse della salute e del benessere dell'uomo e degli animali.

Articolo 4

Ogni Stato membro provvede a vietare gli esperimenti su animali che, ai sensi dell'appendice I della convenzione sul commercio internazionale delle specie di fauna e flora selvatica minacciate di estinzione e dell'allegato C I del regolamento (CEE) n. 3626/82 (1), sono considerati appartenere a specie minacciate, salvo che detti esperimenti siano conformi al regolamento citato e obiettivo dell'esperimento sia:
- la ricerca ai fini della conservazione delle specie di cui trattasi, ovvero

- verifiche medico-biologiche essenziali, allorché la specie di cui trattasi si riveli, eccezionalmente, la sola adatta a tale scopo.

Articolo 5

Per quanto riguarda il trattamento generale e la sistemazione degli animali, gli Stati membri devono provvedere affinché:
a) tutti gli animali da esperimento siano alloggiati e godano di un ambiente, di una certa libertà di movimento, di un'alimentazione, di acqua e di cure adeguate alla loro salute e al loro benessere;
b) qualsiasi limitazione alla possibilità di soddisfare ai bisogni fisiologici e comportamentali di un animale da esperimento sia ridotta al minimo;
c) le condizioni fisiche in cui gli animali da esperimento sono allevati, tenuti o utilizzati siano soggette a controlli giornalieri;
d) il benessere e le condizioni di salute degli animali da esperimento vengano controllati da una persona competente, al fine di evitare danni durevoli, dolore, inutili sofferenze o angoscia;
e) vengano adottate misure intese a correggere tempestivamente difetti o sofferenze eventualmente constatati.
Per l'attuazione delle disposizioni delle lettere a) e b) gli Stati membri s'ispirano alle linee di indirizzo di cui all'allegato II.

Articolo 6

1. Ogni Stato membro designa l'autorità o le autorità cui è demandato il controllo della buona esecuzione delle disposizioni della presente direttiva.
2. Nell'ambito dell'attuazione della presente direttiva gli Stati membri adottano le misure necessarie perché l'autorità designata di cui al paragrafo 1 possa disporre dei pareri di esperti competenti in materia.
(1) GU n. L 384 del 31. 12. 1982, pag. 1.

Articolo 7

1. Gli esperimenti possono essere effettuati soltanto da persone competenti autorizzate o sotto la diretta responsabilità di tali persone, ovvero se il progetto sperimentale o comunque scientifico di cui trattasi è autorizzato in conformità alle disposizioni della legislazione nazionale.
2. Si eviterà di eseguire un esperimento qualora per ottenere il risultato ricercato sia ragionevolmente e praticamente applicabile un altro metodo, scientificamente valido, che non implichi l'impiego di animali.
3. Quando un esperimento è indispensabile, si deve procedere ad un esame attento delle specie e la scelta deve essere eventualmente motivata innanzi all'autorità. Nello scegliere tra esperimenti diversi, devono essere preferiti quelli che richiedono il minor numero di animali, implicano animali con il più basso sviluppo neurologico, causano meno dolore, sofferenza, angoscia o danni durevoli e offrono le maggiori probabilità di risultati soddisfacenti.
È possibile effettuare esperimenti su animali prelevati dall'ambiente naturale soltanto se gli esperimenti su altri animali non rispondono agli scopi dell'esperimento.

4. Tutti gli esperimenti devono essere eseguiti in modo da evitare angoscia e sofferenze o dolore inutili agli animali da esperimento. Gli esperimenti sono soggetti alle disposizioni di cui all'articolo 8. Le misure illustrate all'articolo 9 saranno prese in ogni caso.

Articolo 8

1. Tutti gli esperimenti devono essere effettuati sotto anestesia totale o locale.
2. Il paragrafo 1 non si applica allorché:
a) si ritiene che l'anestesia sia, per l'animale, più traumatica dell'esperimento stesso;
b) l'anestesia è incompatibile con il fine dell'esperimento. In questo caso, si devono adottare appropriate misure legislative e/o amministrative per garantire che nessun esperimento del genere sia effettuato senza necessità.
L'anestesia dovrebbe essere utilizzata nel caso di gravi lesioni che possano causare un forte dolore.
3. Se l'anestesia non è possibile, è necessario ricorrere ad analgesici o ad altri mezzi adeguati così da assicurare, per quanto possibile, che il dolore, la sofferenza, l'angoscia o il danno siano ridotti e l'animale non sia sottoposto a forti dolori, angoscia o sofferenze.
4. Sempreché tale azione sia compatibile con le finalità dell'esperimento, l'animale che, una volta passato l'effetto dell'anestesia, soffra molto deve essere trattato in tempo con degli analgesici o, se questo non è possibile, deve venire immediatamente ucciso con metodi umanitari.

Articolo 9

1. Al termine di ogni esperimento si deciderà se l'animale debba essere mantenuto in vita oppure ucciso con un metodo umanitario; comunque, esso non sarà mantenuto in vita se quand'anche la sua salute fosse ritornata normale sotto tutti gli altri aspetti, è probabile che esso rimanga in permanenti condizioni di sofferenza o di angoscia.
2. Le decisioni di cui al paragrafo 1 devono essere prese da una persona competente, preferibilmente un veterinario.
3. Qualora, al termine di un esperimento:
a) un animale debba essere mantenuto in vita, esso sarà trattato in modo adeguato alle sue condizioni di salute e posto sotto la sorveglianza di un veterinario, o di altra persona competente e tenuto in condizioni conformi alle disposizioni dell'articolo 5. Tuttavia, si potrà derogare alle condizioni fissate al presente paragrafo, qualora, a parere di un veterinario, ciò non comporti alcuna sofferenza per l'animale;
b) un animale non debba essere mantenuto in vita o non possa beneficiare delle disposizioni di cui all'articolo 5 concernenti il suo benessere, esso verrà ucciso al più presto, con un metodo umanitario.

Articolo 10

Gli Stati membri curano che il riutilizzo di animali in esperimenti sia compatibile con le disposizioni delle presente direttiva.
In particolare, un animale non può essere utilizzato più di una volta in esperimenti che comportino forti dolori, angoscia o sofferenze equivalenti.

Articolo 11

A prescindere dalle altre disposizioni della presente direttiva, ove lo rendano necessario i fini legittimi dell'esperimento, l'autorità può consentire che l'animale sia rimesso in libertà, purché abbia la certezza che è stato fatto il possibile per la salvaguardia del benessere di questo, qualora il suo stato di salute lo permetta e non vi sia pericolo per la sanità pubblica e l'ambiente.

Articolo 12

1. Gli Stati membri stabiliscono le procedure per la notifica preventiva all'autorità degli esperimenti medesimi o delle informazioni relative alle persone che procedono a tali esperimenti.
2. Allorché si prevede di sottoporre un animale ad un esperimento che comporta o rischia di comportare per questo un forte dolore che potrebbe protrarsi, l'esperimento deve essere specificamente dichiarato e giustificato presso l'autorità o da questa espressamente autorizzato. L'autorità promuoverà un'azione giudiziaria o amministrativa se non le risulta che l'esperimento sia abbastanza importante per i bisogni essenziali dell'uomo o degli animali.

Articolo 13

1. In base alle richieste di autorizzazione, alle notifiche ricevute, nonché alle relazioni presentate, l'autorità di ogni Stato membro provvede a raccogliere e, per quanto possibile, a pubblicare periodicamente i dati statistici sull'utilizzazione di animili a fini sperimentali per quanto concerne:
a) il numero e le specie di animali utlizzati in esperimenti;
b) il numero di animali, suddivisi in categorie selezionate, utilizzati in esperimenti contemplati dall'articolo 3;
c) il numero di animali, suddivisi in categorie selezionate, utilizzati in esperimenti richiesti dalla legislazione.
2. Gli Stati membri prendono le misure necessarie per garantire il carattere riservato delle informazioni aventi un particolare interesse commerciale che sono comunicate in applicazione della presente direttiva.

Articolo 14

Le persone che effettuano esperimenti o vi prendono parte e le persone che si occupano direttamente o con compiti di controllo di animali utilizzati in esperimenti devono avere un'istruzione e una formazione adeguata.
In particolare, le persone che eseguono gli esperimenti o ne hanno la supervisione devono avere una formazione scientifica attinente alle attività sperimentali di loro competenza e essere in grado di manipolare e curare gli animali di laboratorio; devono inoltre aver dimostrato all'autorità di aver raggiunto un sufficiente livello di formazione in proposito.

Articolo 15

Gli stabilimenti di allevamento e gli stabilimenti fornitori devono essere approvati dall'autorità o registrati presso quest'ultima e soddisfare le condizioni di cui agli articoli 5 e 14, salvo che sia

concessa un'esenzione ai sensi dell'articolo 19, paragrafo 4, o dell'articolo 21. Uno stabilimento fornitore può ricevere animali solo da uno stabilimento di allevamento o da altri stabilimenti fornitori a meno che l'animale sia stato legalmente importato e non si tratti di animale selvatico o randagio. Allo stabilimento fornitore può essere concesso un esonero generale o specifico da quest'ultima disposizione secondo modalità definite dall'autorità.

Articolo 16

L'approvazione o la registrazione di cui all'articolo 15 devono esplicitamente indicare la persona competente che nello stabilimento è incaricata di assicurare direttamente o di organizzare l'assistenza degli animali allevati o alloggiati in tale stabilimento e di far rispettare le disposizioni degli articoli 5 e 14.

Articolo 17

1. Gli stabilimenti di allevamento e gli stabilimenti fornitori sono tenuti a registrare il numero e la specie degli animali venduti o forniti, la data alla quale sono stati venduti o forniti, il nome e l'indirizzo del destinatario nonché il numero e la specie degli animali morti negli stabilimenti stessi.
2. Ogni autorità prescrive i registri che devono essere tenuti e messi a sua disposizione dalla persona responsabile degli stabilimenti di cui al paragrafo 1; tali registri devono essere tenuti per un minimo di tre anni a decorrere dall'ultima registrazione e sono soggetti ad ispezioni periodiche da parte di funzionari dell'autorità.

Articolo 18

1. Ogni cane, gatto o primate non umano che vive in uno stabilimento d'allevamento, fornitore o utilizzatore deve essere dotato, prima dello svezzamento, di un marchio di identificazione individuale nel modo meno doloroso possibile, salvo le eccezioni di cui al paragrafo 3.
2. I cani, i gatti o i primati non umani non contrassegnati che sono portati in uno stabilimento per la prima volta dopo lo svezzamento devono essere contrassegnati non appena possibile.
3. Per i cani, i gatti o i primati non umani non ancora svezzati che vengono trasferiti da uno stabilimento di cui al paragrafo 1 ad un altro, che non sia stato possibile contrassegnare in anticipo, lo stabilimento di destinazione dovrà conservare sino alla marchiatura una documentazione contenente informazioni esaurienti, in particolare l'identità della madre.
4. Nei registri degli stabilimenti devono figurare i dati relativi all'identità e all'origine di tutti i cani, i gatti o i primati non umani presenti.

Articolo 19

1. Gli stabilimenti utilizzatori devono essere registrati presso l'autorità o da questa riconosciuti. Dovranno essere prese disposizioni perché gli stabilimenti utilizzatori dispongano di impianti e attrezzature adeguate alle specie animali utilizzate ed agli esperimenti che vi sono effettuati; la concezione, la costruzione e il funzionamento di questi devono essere tali da garantire che gli esperimenti siano

condotti nel modo più appropriato possibile, al fine di ottenere risultati coerenti con il minimo numero possibile di animali ed il minimo di dolore, sofferenza, angoscia o danni durevoli.
2. In ogni stabilimento utilizzatore:
a) deve (devono) essere designata(e) la persona (le persone) amministrativamente responsabile(i) dell'assistenza degli animali e del funzionamento delle attrezzature;
b) deve essere disponibile un numero sufficiente di persone qualificate;
c) è necessario prevedere opportune disposizioni perché si avvalga della consulenza e dell'assistenza veterinaria;
d) si deve incaricare un veterinario o altra persona competente di fornire consulenza sul benessere degli animali.
3. Su autorizzazione dell'autorità, gli esperimenti possono essere eseguiti fuori degli stabilimenti utilizzatori.
4. Negli stabilimenti utilizzatori possono essere utilizzati soltanto gli animali provenienti da stabilimenti d'allevamento o fornitori, a meno che sia stato ottenuto un esonero generale o speciale conformemente alle disposizioni definite dall'autorità. Per quanto possibile devono essere usati animali d'allevamento. Gli animali randagi delle specie domestiche non devono essere usati negli esperimenti. L'esonero generale concesso alle condizioni di cui al presente paragrafo non può essere esteso ai cani e gatti randagi.
5. Gli stabilimenti utilizzatori devono tenere dei registri di tutti gli animali utilizzati e, su richiesta, metterli a disposizione delle autorità. In particolare, i registri devono indicare il numero e la specie di tutti gli animali acquistati, la provenienza e la data del loro arrivo. I registri devono essere tenuti per almeno tre anni e presentati all'autorità che ne faccia richiesta. Gli stabilimenti utilizzatori sono periodicamente ispezionati da rappresentanti dell'autorità.

Articolo 20

Gli stabilimenti utilizzatori che allevano animali per esperimenti da eseguire nei loro locali devono richiedere una sola registrazione o approvazione ai sensi degli articoli 15 e 19. Sono però tenuti a ottemperare alle disposizioni della direttiva relative agli stabilimenti di allevamento e agli stabilimenti utilizzatori.

Articolo 21

Gli animali appartenenti alle specie elencate nell'allegato I e da utilizzare in esperimenti devono essere animali da allevamento, a meno che sia stato ottenuto un esonero generale o speciale conformemente alle disposizioni fissate dall'autorità.

Articolo 22

1. Per evitare inutili ripetizioni degli esperimenti destinati ad ottemperare a disposizioni legislative nazionali o comu-
nitarie relative alla salute od alla sicurezza, gli Stati membri riconoscono per quanto è possibile la validità dei dati risultanti dagli esperimenti eseguiti nel territorio di un altro Stato membro, a meno che non siano necessarie ulteriori prove per proteggere la pubblica salute e la sicurezza.
2. A tal fine gli Stati membri, ove sia possibile e fatte salve le disposizioni delle vigenti direttive comunitarie, forniscono alla Commissione informazioni sulle rispettive legislazioni e pratiche

amministrative relative agli esperimenti su animali, ivi compresi gli obblighi cui ottemperare prima di commercializzare i prodotti, nonché informazioni di fatto su tutti gli esperimenti svolti nei rispettivi territori e sulle autorizzazioni o su ogni altro elemento di ordine amministrativo concernente detti esperimenti.
3. La Commissione istituisce un comitato consultivo permamente, nel cui ambito saranno rappresentati gli Stati membri, il quale assisterà la Commissione nell'organizzazione dello scambio di informazioni appropriate, osservando la necessaria riservatezza, e che assisterà anche la Commissione per quanto riguarda gli altri problemi sollevati dall'applicazione della presente direttiva.

Articolo 23

1. La Commissione e gli Stati membri dovrebbero incoraggiare la ricerca intesa a sviluppare e rendere più efficaci tecniche alternative atte a fornire lo stesso livello d'informazione degli esperimenti su animali ma che prevedano l'utilizzo di un minor numero di animali o comportino procedimenti meno dolorosi e prendono tutte le misure che ritengono opportune per favorire la ricerca in questo settore. La Commissione e gli Stati membri seguono l'evoluzione dei metodi sperimentali.
2. Prima della fine del 1987 la Commissione farà una relazione sulla possibilità di modificare le prove e gli orientamenti stabiliti dalla legislazione comunitaria in vigore, tenendo conto degli obiettivi di cui al paragrafo 1.

Articolo 24

La presente direttiva non limita il diritto degli Stati membri di applicare, o di adottare, misure più rigide per la protezione degli animali utilizzati a fini sperimentali o per il controllo e la limitazione dell'uso degli animali in esperimenti. In particolare, gli Stati membri possono esigere un'autorizzazione preliminare per esperimenti o programmi di lavoro notificati in base alle disposizioni dell'articolo 12, paragrafo 1.

Articolo 25

1. Gli Stati membri prendono le misure necessarie per conformarsi alla presente direttiva entro il 24 novembre 1989. Essi ne informano immediatamente la Commissione.
2. Gli Stati membri comunicano alla Commissione le disposizioni di diritto interno che essi adottano nel settore disciplinato dalla presente direttiva.

Articolo 26

A intervalli regolari, non superiori a tre anni, e per la prima volta cinque anni dopo la notifica della presente direttiva, gli Stati membri informano la Commissione delle misure adottate in materia e presentano un compendio delle informazioni raccolte conformemente all'articolo 13. La Commissione redige una relazione da presentare al Consiglio e al Parlamento europeo.

Articolo 27

Gli Stati membri sono destinatari della presente direttiva.

Fatto a Bruxelles, addì 24 novembre 1986.
Per il Consiglio
Il Presidente
W. WALDEGRAVE
