# Supplementary material for: Localizing Tortoise Nests by Neural Networks
Source: PLoS One. 2016 Mar 17;11(3):e0151168. doi: 10.1371/journal.pone.0151168 (PMC4795789; doi:10.1371/journal.pone.0151168)
Supplement: S2 File — (PDF) [file pone.0151168.s002.pdf]

UNIVERSITÀ DI PISA
CENTRO DI ATENEO
MUSEO DI STORIA NATURALE

MUSEO DI STORIA NATURALE

UNIVERSITA' DI PISA
Codice AOO: CAL
Num. Prot.: 0000529 / 2015
Data: 22/05/2015
PROTOCOLLO IN **USCITA**

Calci, May 22, 2015

As director of the Museum of Natural History of the University of Pisa, which is manager, together with the "Unione dei Comuni Montana Colline Metallifere", of the "Protection Center for Mediterranean Tortoises" in Massa Marittima, Tuscany, Italy, I confirm that the collection of accelerometer data from nesting tortoises of the Protection Center, during the period May 2012 – July 2012, was authorized by the Museum of Natural History.

Roberto Barbuti
Director

Via Roma, 79 – 56011 – Calci – Pisa – Italia
Centralino tel. e fax (+39) 050 2212990050-937092; Direzione e Segreteria tel. (+39) 050 2212975 –
Informazioni e visite tel. e fax (+39) 050 2212970 – Partita I.V.A. 00286820501 – Codice fiscale 80003670504
