# Supplementary material for: Localizing Tortoise Nests by Neural Networks
Source: PLoS One. 2016 Mar 17;11(3):e0151168. doi: 10.1371/journal.pone.0151168 (PMC4795789; doi:10.1371/journal.pone.0151168)
Supplement: S3 File — (PDF) [file pone.0151168.s003.pdf]

REGIONE TOSCANA

GIUNTA REGIONALE

ESTRATTO DAL VERBALE DELLA SEDUTA DEL 04-06-2012 *(punto N 9)*

Delibera *N 479* *del 04-06-2012*

Proponente

ANNA RITA BRAMERINI

DIREZIONE GENERALE POLITICHE TERRITORIALI, AMBIENTALI E PER LA MOBILITA'

Pubblicita'/Pubblicazione Atto soggetto a pubblicazione integrale (PBURT/BD)

Dirigente Responsabile PAOLO MATINA

Estensore ANDREA CASADIO

Oggetto

L.R. 56/00, art. 9 e 12. Riconoscimento come centro per la conservazione ex situ della fauna del Centro di Protezione e Custodia di Erpetofauna Selvatica in loc. Malfatto in Comune di Massa marittima (GR)

Presenti

ENRICO ROSSI	SALVATORE ALLOCCA	ANNA RITA BRAMERINI
LUCA CECCOBAO	RICCARDO NENCINI	GIANNI SALVADORI
CRISTINA SCALETTI	GIANFRANCO SIMONCINI	STELLA TARGETTI
LUIGI MARRONI		

Assenti

ANNA MARSON

STRUTTURE INTERESSATE

Tipo	*Denominazione*
Direzione Generale	DIREZIONE GENERALE POLITICHE TERRITORIALI, AMBIENTALI E PER LA MOBILITA'

LA GIUNTA REGIONALE

Vista la Direttiva 92/43/CEE “Habitat” del Consiglio delle Comunità Europee del 21 maggio 1992 concernente la conservazione degli habitat naturali e seminaturali e della flora e della fauna selvatiche;

Vista la Direttiva 2009/147/CE del Parlamento europeo e del Consiglio del 30 novembre 2009 concernente la conservazione degli uccelli selvatici in abrogazione della precedente Direttiva 79/409/CEE del Consiglio del 2 aprile 1979;

Visti gli articoli 12 e 13 della Direttiva 92/43/CEE (Habitat) che prevedono un regime di tutela per le specie animali e vegetali a rischio di estinzione di cui all’allegato IV della Direttiva stessa, lettere a) e b);

Visto l’art. 14 della sopra citata Direttiva riguardante specifiche misure che possono essere messe in atto da parte degli Stati membri per il mantenimento in uno stato di conservazione soddisfacente delle specie della fauna e della flora selvatiche di interesse comunitario di cui all’allegato V della Direttiva;

Visto il Decreto del Presidente della Repubblica 8 settembre 1997, n. 357, (Regolamento recante attuazione della Direttiva 92/43/CEE relativa alla conservazione degli habitat naturali e seminaturali, nonché della flora e della fauna selvatiche) così come modificato dal D.P.R. 120/2003;

Visti gli articoli 10 e 11 del D.P.R. 357/97 che, conformemente a quanto previsto dalla sopra citata Direttiva, indicano misure che possono essere adottate al fine di tutelare le specie di fauna e di flora selvatiche il cui prelievo e sfruttamento possono richiedere azioni specifiche per il loro mantenimento in uno stato di conservazione soddisfacente;

Vista la Legge Regionale 6 aprile 2000 n. 56 avente per oggetto “Norme per la conservazione e la tutela degli habitat naturali e seminaturali, della flora e della fauna selvatiche” e successive modificazioni ed integrazioni con cui la Regione Toscana ha dato attuazione alla Direttiva Habitat ed al D.P.R. 357/97 di recepimento sopra indicato;

Visto l’articolo 9 della predetta legge avente per oggetto “Centri di conservazione della fauna e della flora selvatiche” in cui viene stabilito che la Regione provvede al riconoscimento di Centri per la conservazione, la riproduzione, il recupero ed il ricovero di specie animali e vegetali di interesse regionale e definisce i requisiti strutturali, organizzativi e strumentali degli stessi anche ai fini dell’erogazione di eventuali finanziamenti;

Preso atto che, con Deliberazione di Giunta Regionale n. 1175 del 22 novembre 2004 inerente “LR 56/00 - Art. 12 comma 1 lettera E - Definizione dei requisiti strutturali dei centri previsti dall’art. 9, nonchè dei requisiti organizzativi e strutturali dei soggetti gestori dei centri stessi” la Regione Toscana ha provveduto a definire i suddetti requisiti strutturali, organizzativi e strumentali che i centri devono avere per essere riconosciuti come centri per la conservazione ex situ di interesse regionale;

Visti gli allegati A, B e C della L.R. 56/2000 in cui sono elencate le specie animali e vegetali di interesse conservazionistico;

Vista la nota inviata dalla Comunità Montana Colline Metallifere in data 05/08/11 (n.prot.6053 – 450), nella quale si faceva richiesta di riconoscimento del Centro di protezione e custodia di erpetofauna selvatica situato in Loc. Malfatto – Comune di Massa Marittima quale Centro per la Conservazione Ex situ della fauna di interesse regionale (CESFA), ai sensi dell'art.12 della LR 56/00 e della DGR 1175/04 per i seguenti gruppi tassonomici:
- Anfibi, Rettili (compresi quelli ritenuti pericolosi in base al decreto del Ministero dell'Ambiente 19/4/96) e invertebrati;

Vista la documentazione allegata alla sopra citata nota e conservata agli atti del competente settore con la quale vengono descritti i requisiti previsti dall'Allegato A alla DGR 1175/04;

Vista la nota inviata dal settore tutela e valorizzazione risorse ambientali in data 22/03/2012 (n.prot.AOO-GRT-0084950/P.130.20) con la quale veniva richiesta ai competenti uffici della Provincia di Grosseto la formulazione di un parere tecnico sul possesso dei requisiti da parte del Centro situato in Loc. Malfatto e sull'eventuale suo riconoscimento quale centro di interesse regionale ai sensi della citata normativa;

Vista la nota inviata dall'area Ambiente e Conservazione della Natura della provincia di Grosseto in data 27/04/12 (n. prot. AOO-GRT 122584/P.130.30) con la quale è stato comunicato il parere positivo al riconoscimento come CESFA del Centro situato in Loc. Malfatto;

Preso atto che, a seguito dell'analisi di detta documentazione da parte del settore regionale competente Tutela e valorizzazione risorse ambientali e dello specifico sopralluogo che il medesimo ha realizzato in data 13/01/2012, presso il centro situato in località Malfatto, al fine di valutare lo stato dei luoghi, è stata accertata la sussistenza nel medesimo Centro dei requisiti previsti dall'Allegato A della DGR 1175/04 per il suo riconoscimento quale CESFA , ai sensi dell'art.12 della LR 56/00;

Visto il parere favorevole espresso dalla Consulta tecnica per le aree protette e la biodiversità sulla sopra citata richiesta di riconoscimento riportato nel verbale della seduta del 29 Febbraio 2012 e conservato agli atti dal Settore Tutela e valorizzazione risorse ambientali;

Considerata la necessità di dare attuazione alla LR 56/2000 relativamente al riconoscimento di Centri di conservazione di interesse regionale che rappresentano un elemento fondamentale per il mantenimento ed il ripristino delle popolazioni di flora e fauna a rischio di estinzione in uno stato di conservazione soddisfacente e per il perseguimento di una efficace strategia regionale nel campo della conservazione delle specie animali e vegetali;

A voti unanimi

DELIBERA

1. di riconoscere come Centro per la Conservazione Ex Situ della Fauna (CESFA), ai sensi dell'art 9 e 12 della LR 56/00, il Centro di protezione e custodia di erpetofauna selvatica, situato in località Malfatto, (Massa Marittima - Grosseto) in quanto in possesso dei requisiti previsti dall'Allegato A alla DGR 1175/04 per i seguenti gruppi tassonomici:
   - Anfibi, Rettili (compresi quelli ritenuti pericolosi in base al decreto del Ministero dell'Ambiente 19/4/96) e invertebrati;

2. di dare mandato ai competenti uffici della Giunta regionale di trasmettere copia della presente deliberazione alla Amministrazione Provinciale di Grosseto affinché ne tenga conto nello svolgimento degli adempimenti di propria competenza finalizzati a dare attuazione all’art. 9 della LR 56/2000.

Il presente provvedimento, soggetto a pubblicazione ai sensi dell’art.5 comma 1, lett. f) e dell’art. 18, comma 2, lett. a) della L.R. 23/2007, in quanto conclusivo di procedimento amministrativo, è pubblicato integralmente sul BURT e sulla banca dati degli atti amministrativi della Giunta Regionale.

SEGRETERIA DELLA GIUNTA
IL DIRETTORE GENERALE
ANTONIO DAVIDE BARRETTA

Il Dirigente responsabile
PAOLO MATINA

Il Direttore Generale
RICCARDO BARACCO
