# Supplementary material for: Localizing Tortoise Nests by Neural Networks
Source: PLoS One. 2016 Mar 17;11(3):e0151168. doi: 10.1371/journal.pone.0151168 (PMC4795789; doi:10.1371/journal.pone.0151168)
Supplement: S4 File — (PDF) [file pone.0151168.s004.pdf]

Unione di Comuni montana Colline Metallifere

Comuni di Massa Marittima, Monterotondo Marittimo, Montieri

05 GIU 2015

Massa Marittima, Ma[illegible] 2015

UCM Colline Metallifere
Protocollo Generale: Partenza
n° 0003998 del 05-06-2015 ore 11:04:01
Classificazione 2.9
UCR:SEGRET ARIATO GENERALE

As president of the "Unione dei Comuni Montana Colline Metallifere", which is manager, together with the Museum of Natural History of the University of Pisa, of the "Protection Center for Mediterranean Tortoises" in Massa Marittima, Tuscany, Italy, I confirm that the collection of accelerometer data from nesting tortoises of the Protection Center, during the period May 2012 – July 2012, was authorized by the Unione dei Comuni Montana Colline Metallifere.

UNIONE DI COMUNI MONTANA COLLINE METALLIFERE - SEDE IN MASSA MARITTIMA

Marcello Giuntini
President

Piazza Dante Alighieri, 4 – 58024 Massa Marittima (GR) - C.F. 92074720530 - Partita IVA 01506330537
Telefono 0566 906111 - Fax 0566 903530 sito web: www.unionecomunicollinemetallifere.it
e-mail: info@unionecomunicollinemetallifere.it
PEC (posta elettronica certificata): unionecomuni.collinemetallifere@postacert.toscana.it
